# EMeth: An EM algorithm for cell type decomposition based on DNA methylation data

**DOI:** 10.1038/s41598-021-84864-9

**Published:** 2021-03-11

**Authors:** Hanyu Zhang, Ruoyi Cai, James Dai, Wei Sun

**Affiliations:** 1 Department of Statistics, University of Washington, Seattle, USA; 2 Department of Biostatistics, University of Washington, Seattle, USA; 3 Public Health Science Division, Fred Hutchinson Cancer Research Center, Seattle, USA; 4 Department of Biostatistics, University of North Carolina, Chapel Hill, USA

**Keywords:** Cancer, Computational biology and bioinformatics, Statistics

## Abstract

We introduce a new computational method named EMeth to estimate cell type proportions using DNA methylation data. EMeth is a reference-based method that requires cell type-specific DNA methylation data from relevant cell types. EMeth improves on the existing reference-based methods by detecting the CpGs whose DNA methylation are inconsistent with the deconvolution model and reducing their contributions to cell type decomposition. Another novel feature of EMeth is that it allows a cell type with known proportions but unknown reference and estimates its methylation. This is motivated by the case of studying methylation in tumor cells while bulk tumor samples include tumor cells as well as other cell types such as infiltrating immune cells, and tumor cell proportion can be estimated by copy number data. We demonstrate that EMeth delivers more accurate estimates of cell type proportions than several other methods using simulated data and in silico mixtures. Applications in cancer studies show that the proportions of T regulatory cells estimated by DNA methylation have expected associations with mutation load and survival time, while the estimates from gene expression miss such associations.

## Introduction

Almost all bulk tissue samples are composed of multiple cell types. Cell type proportion estimates can be very valuable for -omic data analysis or clinical studies. For example, accounting for cell type proportion variation is critical in Epigenome Wide Association Studies^1^. Another example is that immune cell composition in tumor samples can predict response to checkpoint inhibitor immunotherapy^2^. This is likely because checkpoint inhibitors work by reinvigorating a pre-existing tumor immune response, which can be characterized by the cell types and the extent of immune cell infiltration in tumor samples^3^.

Both gene expression and DNA methylation vary across cell types and can be used to estimate cell type composition. An advantage to estimate cell type composition using DNA methylation instead of gene expression is that DNA methylation is usually more stable than gene expression and it is easier to measure in formalin-fixed paraffin-embedded or FFPE tissues^4^, which is the most commonly used forms to store tissue samples. More computational methods have been developed for cell type deconvolution using gene expression data. Many lessons learned from gene-expression-based deconvolution methods are useful for DNA-methylation-based deconvolution, and thus we will briefly review the earlier works using either gene expression or DNA methylation data.

CIBERSORT/CIBERSORTx is among the most popular methods for gene-expression-based deconvolution and it delivers comparable or more accurate estimates of cell type composition than many earlier methods^5,6^. The core of CIBERSORT is a support vector regression where the response variable is the gene expression from bulk tissues and each covariate corresponds to the gene expression from one cell type, which are usually estimated from external reference samples. The good performance of CIBERSORT is in part due to the fact that the objective function of a support vector regression is robust to the noise in the data. In contrast, some commonly used objective functions (e.g., the summation of squared residuals of a linear regression) are sensitive to outliers. We will include support vector regression as one of the competing methods for DNA-methylation-based deconvolution in this study.

Another recent method for gene expression-based deconvolution is ICeD-T^7^, which models gene expression by a log-normal distribution. This model choice allows the decomposition in linear-scale^8^ and evaluation of the loss function in log-scale where gene expression variance is much more stable than in linear scale. ICeD-T employs a mixture of regression model to identify those genes whose expression in a tissue sample is inconsistent with the deconvolution model and it down-weighs the contribution of those genes for cell type deconvolution. Our EMeth method is motivated by the ICeD-T method and adopts a similar mixture of regression approach.

In general, there are two classes of methods studying cell type decomposition using DNA methylation data: reference-based methods vs. reference-free methods. Reference-free methods do not require the reference of cell type-specific DNA methylation^9–11^. Their main goal is to account for the variation of cell type composition in the association analysis of DNA methylation^12^, and the latent factors estimated from these methods are often linear combinations of cell type proportions rather than the cell type proportions themselves. The reference-based methods, which require prior knowledge of cell type-specific DNA methylation, can estimate the proportion of each cell type. Our EMeth method is a reference-based method.

A few reference-based methods have been developed in a regression framework where the response variable is the DNA methylation of multiple CpGs in a tissue sample and each covariate is the DNA methylation of these CpGs in a cell type. Houseman et al.^13^ proposed a linear regression method with quadratic programming to impose the constraint that the regression coefficients are none-negative. Teschendorff et al.^14^ proposed a method Epidish that replaces linear regression with robust linear regression (R function MASS/rlm) that uses a weighted loss function so that the data points with larger residuals have smaller weights. Motivated by the success of CIBERSORT in gene expression decomposition, Teschendorff et al.^14^ and Chakravarthy et al.^15^ employed support vector regression to estimate cell type composition using DNA methylation data.

A limitation of reference-based methods is that for some CpGs, the DNA methylation in the reference samples may not accurately capture the cell type-specific DNA methylation in a tissue sample. Another limitation is that reference may not be available for all cell types. For example, when studying tumor immune microenvironment, we often have the reference for immune cell types, but not for tumor cells. Chakravarthy et al.^15^ have used the DNA methylation derived from tumor cell lines as the reference for tumor cells, which may not be ideal for many cancer types where there are considerable differences between cell lines and bulk tumor samples.

To overcome these two limitations, we propose a new method for cell type decomposition using DNA methylation data. We name our method *EMeth*, since it uses an EM (Expectation-Maximization) algorithm for parameter estimation and it is applied on DNA methylation data. To overcome the challenge of inaccurate reference for some CpGs, EMeth models the observed DNA methylation of each CpG by a mixture distribution with one component for regular/consistent CpGs and the other component for aberrant CpGs whose DNA methylation are inconsistent with what is expected from the deconvolution model. EMeth automatically down-weighs the contributions of the aberrant CpGs on cell type deconvolution. To overcome the second challenge of unknown reference, EMeth includes a special cell type without methylation reference, but with known cell type proportions. This is motivated by cancer studies where tumor purity can be estimated from DNA copy number data, or even methylation data itself^16^. EMeth estimates the DNA methylation of this special cell type.

We evaluated EMeth and four competing methods (linear regression, robust linear regression, quadratic programming, and support vector regression) by two sets of studies where DNA methylation were generated by simulation or in silico mixture of observed cell type-specific DNA methylation. Our results demonstrate that EMeth has better performance than all the competing methods. Then we applied EMeth on four cancer types that have relatively higher mutation burdens, hence higher likelihood of immune infiltration^17^: colon adenocarcinoma (COAD), lung adenocarcinoma (LUAD), lung squamous cell carcinoma (LUSC), and skin cutaneous melanoma (SKCM). We found that cell type proportions estimated by EMeth have good consistency with the estimates from gene expression data for CD8T and B cells, but correlated poorly for T regulatory cells (Tregs). The Tregs proportion estimates by DNA methylation were negatively associated with mutation load and survival time, consistent with the functional roles of Tregs. Such associations were missed by the Treg proportion estimates by gene expression. Therefore, the cell type proportion estimates by DNA methylation provide some helpful additions to those estimates by gene expression.

## Results

### Method overview and study design

DNA methylation of a CpG site can be measured by a beta-value or an M-value. A beta-value is between 0 and 1 and it quantifies the proportion of DNA molecules in which this CpG is methylated. An M-value is the logit transformation of a beta-value. Appropriate decomposition should be performed using beta-values^13^.

The input data of EMeth include the methylation data (beta-values) from bulk tissue samples and the reference data of cell type-specific DNA methylation in a pre-defined set of CpGs. For each bulk sample, these CpGs can be divided into two groups: the consistent (or aberrant) CpGs on which the DNA methylation in the bulk sample is consistent (or inconsistent) with what is expected from the deconvolution model. Note that the set of aberrant CpGs may vary across bulk tissue samples. EMeth models the DNA methylation data in a tissue sample by a normal mixture of regression model with two components, designed for consistent and aberrant CpGs, respectively. We assume the aberrant component has a larger variance, which can be estimated from the data and such larger variance automatically down-weighs the contribution of the aberrant CpGs for cell type deconvolution. Within each mixture component, EMeth assumes the mean value of DNA methylation is a weighted summation of cell type-specific DNA methylation where the weights are cell type proportions. A standard EM algorithm can be used to estimate the parameters of this mixture of regression model. From our exploratory analysis, we found the estimation may be unstable when two or more cell types have highly correlated cell type-specific DNA methylation. To alleviate this problem, we added a ridge penalty on cell type compositions and incorporated it in the likelihood function for EMeth.

EMeth allows a special cell type of which the cell type proportions in tissue samples are known but the corresponding cell type-specific methylation reference is unknown. This is often the case for tumor tissues where tumor purity is known but DNA methylation in tumor cells is unknown. The methylation level of this special cell type can be estimated by borrowing information across tissue samples. For each CpG, the expected contribution of this special cell type to the observed methylation is proportional to the product of its proportion in a tissue sample and its methylation. Therefore, its methylation can be estimated by a regression using the methylation data of this CpG across tissue samples, where the response variable is the observed methylation, and the covariate is the proportion of this cell type. See the Methods Section for more details.

Among the outputs of EMeth, most users may be interested in the estimates of cell type proportions and the DNA methylation in the special cell type. Though we also provide the estimate of aberrant probability for each CpG in each bulk sample, which can be informative for CpGs selection by other deconvolution methods that do automatically weigh each CpG.

EMeth is implemented in an open-source R package, freely available at https://github.com/Hanyuz1996/EMeth. More details of EMeth are presented in the Methods Section, and Section 1 of the Supplementary Materials.

We compared EMeth versus the following four methods.

1. Ordinary linear regression that minimizes residual sum squares of the model fit (least squares or **LS**).
2. Support vector regression (**SVR**) with linear kernel. This is the method used by CIBERSORT/CIBERSORTx^5,15^. There is a cost parameter in svr that balances the model fit and penalty. The default cost parameter does not work well, and we choose it by cross-validation.
3. Robust linear regression (**RLS**) with Huber loss. This is the method used by Epidish^14^.
4. Linear regression with constraints that the regression coefficients are non-negative, solved by quadratic programming (**QP**). This is the method used by Houseman et al.^13^.

For all these methods, the regression coefficients are the estimates of cell type proportions. In the first three methods, a coefficient is set to 0 if it is smaller than 0. Then we re-normalize the coefficients such that they add up to one for each sample. All these four methods are based on a linear mean structure of methylation but do not consider variance structure. In contrast, EMeth uses the variance to detect aberrant CpGs. These methods also do not allow a special cell type with known proportions but unknown methylation.

### Evaluation of different methods using simulated data

This simulation study is based on cell type-specific DNA methylation data from seven types of immune cells (CD4T, CD8T, B cells, Natural Killer cells, Neutrophils, Monocytes, and T Regulatory Cells). In order to generate this cell type-specific reference dataset, we combined cell type-specific DNA methylation data measured by Illumina 450k arrays from six studies^18–23^. The raw data were processed to remove batch effects due to different data sources, see Section 2 of the Supplementary Materials for details.

We first estimated the mean methylation level per CpG and per cell type using this cell type-specific reference dataset. For the special cell type without reference, the mean methylation of each CpG was simulated independently across CpGs from a uniform (0,1) distribution. Then we randomly simulated cell type proportions for each tissue sample and calculated the mean methylation of each CpG in each tissue sample by the weighted summation of cell type-specific methylation, where the weights were cell type proportions. Finally, we simulated methylation in each tissue sample by a normal distribution with these mean values and variances calculated based on a mean-variance relation of the binomial distribution (see Methods Section for more details). For each sample, the proportion of aberrant CpGs was sampled from a uniform distribution in the range of 5% to 15%. We set the number of bulk tissue samples to be 100 for all simulations and selected 946 CpGs to construct the reference data. The details of CpG selection can be found in Section 3 of the Supplementary Materials.

The key parameters in this simulation include (1) a variance parameter $\sigma _c^2$, which represents the noise level in the consistent CpGs; and (2) the ratio of the variance of aberrant CpGs vs. consistent CpGs: $\lambda =\sigma _a^2/\sigma _c^2$. The value of $\sigma _c^2$ is based on the estimates from four cancer types that are part of The Cancer Genome Atlas (TCGA) study. We evaluate the performance of different methods by rooted mean square error (RMSE). Specifically, we first computed the difference of the estimated proportions and the true proportions for each cell type, and then computed the square root of the average of the squared difference. For each method, the average was taken across all cell types and all samples. More details of simulation setup are provided in Section 3.1 of the Supplementary Materials.

EMeth always reaches the lowest RMSE in all simulation settings (Table 1). Among the four benchmark algorithms, SVR and RLS have similar performance and both have smaller RMSE than QP, which has slightly smaller RMSE than LR. When $\lambda = \sigma _a^2/\sigma _c^2$ increases from 2 to 100, the performance of all the four competing methods become worse, which is expected since there is more noise in the data; while in contrast, the performance of EMeth becomes better. This is because larger $\lambda$ makes it easier for EMeth to separate the aberrant CpGs from the consistent ones and thus appropriately down-weighs their contributions to the final estimates. For example, for the middle level of $\sigma _c^2=0.00221$ (corresponding to the $\sigma _c^2$ estimate from lung adenocarcinoma (LUAD) patients) and $\lambda =2$, EMeth reduces the RMSE of SVR by 31% (from 0.466 to 0.322). For the same $\sigma _c^2$ but with larger $\lambda =100$, EMeth reduces the RMSE of SVR by 36% (from 0.486 to 0.310). We also conducted additional simulations where the proportion of aberrant CpG probes varies from 5 to 35% and reached the same conclusion that EMeth consistently outperforms all the other methods (Supplementary Table S3).

**Table 1:** Estimation results from a simulation study. Cell type proportion estimation accuracy, evaluated in terms of rooted mean square error (RMSE) for 18 simulation settings, with six values of $\sigma _c^2$, the noise level for consistent CpGs, and 3 values of $\lambda$, the ratio of the noise level between the aberrant CpGs and the consistent CpGs. Four of the six values of $\sigma _c^2$ are selected according to estimation from four cancer types of the TCGA study.

$\sigma _c^2$	$\lambda =\sigma _a^2/\sigma _c^2$	EMeth	SVR	LS	RLS	QP
0.004	2	0.0268	0.0412	0.0806	0.0384	0.0771
	10	0.0261	0.0418	0.0833	0.0400	0.0794
	100	0.0263	0.0429	0.0907	0.0423	0.0856
0.00298 (LUSC)	2	0.0264	0.0403	0.0781	0.0362	0.0749
	10	0.0262	0.0410	0.0807	0.0376	0.0771
	100	0.0258	0.0419	0.0878	0.0397	0.0832
0.00221 (LUAD)	2	0.0259	0.0397	0.0758	0.0344	0.0730
	10	0.0258	0.04021	0.0782	0.0356	0.0750
	100	0.0253	0.0411	0.0850	0.0374	0.0808
0.00141 (SKCM)	2	0.0249	0.0387	0.0727	0.0322	0.0703
	10	0.0251	0.0309	0.0747	0.0331	0.0720
	100	0.0251	0.0399	0.00810	0.0347	0.0774
0.00118 (COAD)	2	0.0248	0.0384	0.0716	0.0315	0.0693
	10	0.0250	0.0388	0.0735	0.0324	0.0710
	100	0.0248	0.0396	0.0795	0.0338	0.0762
0.001	2	0.0245	0.0382	0.0706	0.0309	0.0684
	10	0.0248	0.0386	0.0724	0.0317	0.0700
	100	0.0249	0.0393	0.0782	0.0330	0.0750

**Figure 1:**
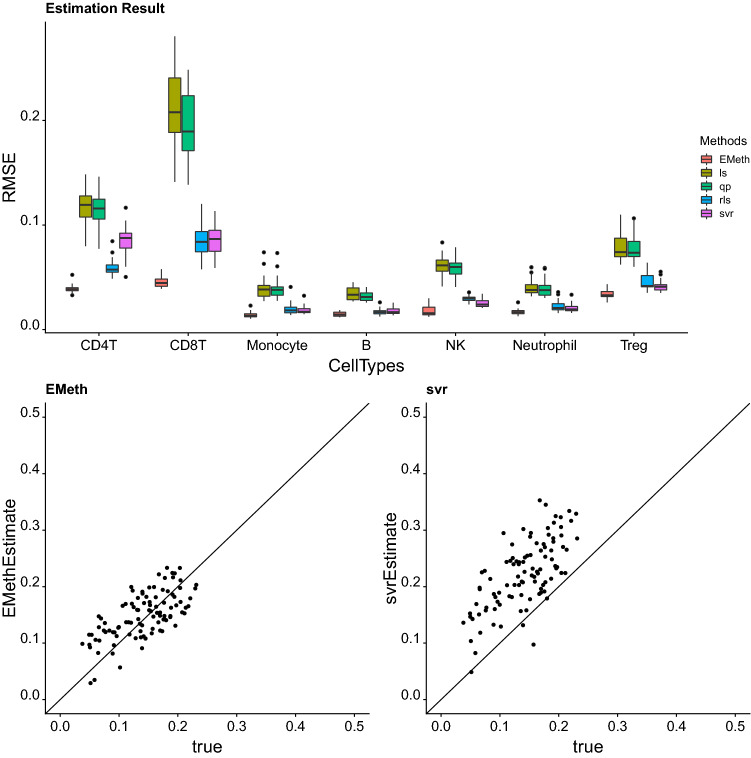
Evaluation of different methods using simulated data. The upper panel shows the RMSE in 25 repetitions of all five methods in one group of simulation parameters ($\sigma _c^2 = 0.004$, $\lambda = 10$). We compare the RMSE for each cell type separately. Under this setting, EMeth dominates the result in all cell types. Two panels in the bottom panel display estimates of CD4T proportions by EMeth and SVRs for 100 samples. In this case, both methods have reasonable performances, though SVR has a systematic bias. These plots were generated using ggplot2 version 3.3.1^24^.

We also investigated the cell proportion estimates for each cell type separately. EMeth performs the best for all the cell types in most simulation settings (Fig. 1 and Figures S5-S22 in Section 3 of the Supplementary Materials). The estimation accuracy varies a lot across cell types. For some cell types all the methods provide accurate estimates. For example, the RMSE can be as low as 0.01 for monocyte by all the methods. This low RMSE is not an artifact of low cell type proportions since the expected proportions of all the cell types are the same in this simulation. In contrast, the RMSE can be relatively high for CD4T or CD8T. RMSE for CD8T can be as high as 0.05 for EMeth, and higher than 0.05 for all the other four methods. The difficulty in estimating the proportions of CD4T and CD8T is due to the high level of similarity of DNA methylation for these two cell types. In the reference data, the correlation between the average methylation of CD4T and CD8T can reach 0.96. The ridge penalty in our model helps improve the robustness against this collinearity and leads to more accurate estimates.

In addition to cell type proportions, EMeth also estimates the DNA methylation in the special cell type without reference. Our simulation results show that the EMeth estimates are reasonably accurate. The correlations between the estimates and the true values are around or above 0.7 in most cases (Table S2 in the Supplementary Materials).

### Evaluation of different methods using in silico mixtures of cell type-specific DNA methylation data

Next, we evaluated different methods by directly mixing individual-specific and cell type-specific DNA methylation data to generate pseudo tissue samples. This approach captures the variation of cell type-specific DNA methylation across individuals and does not rely on any distribution assumption. To this end, we used the methylation and gene expression data in three human immune cell types (Monocytes, Neutrophils and naive CD4 T cells) from blood samples of 197 individuals, which were generated as part of the BLUEPRINT project^25^. This is a very unique and valuable dataset because it includes cell type-specific omic data in a large number of individuals. We selected the 124 individuals with complete gene expression and DNA methylation data for all three cell types for this evaluation. We compared cell type decomposition based on DNA methylation data by EMeth and four other methods as well as cell type decomposition using gene expression data by CIBERSORTx^26^. We did not consider the special cell type with unknown methylation in this in silico mixing dataset.

For each individual, we simulated a mixture by linearly combining cell type-specific data, followed by adding Gaussian noise in M-value scale for methylation data and in log-scale for expression data. The only parameter in this simulation study is the variance of the Gaussian noise. Since we use the same mixture proportions on both methylation and gene expression data, we are able to compare the cell type proportion estimates from these two types of data. We used the data from 56 individuals to construct the reference data and the remaining 68 individuals to generate mixture samples. We added the same level of noise to both expression and methylation data. See Section 4 of the Supplementary Materials for more details.

**Figure 2:**
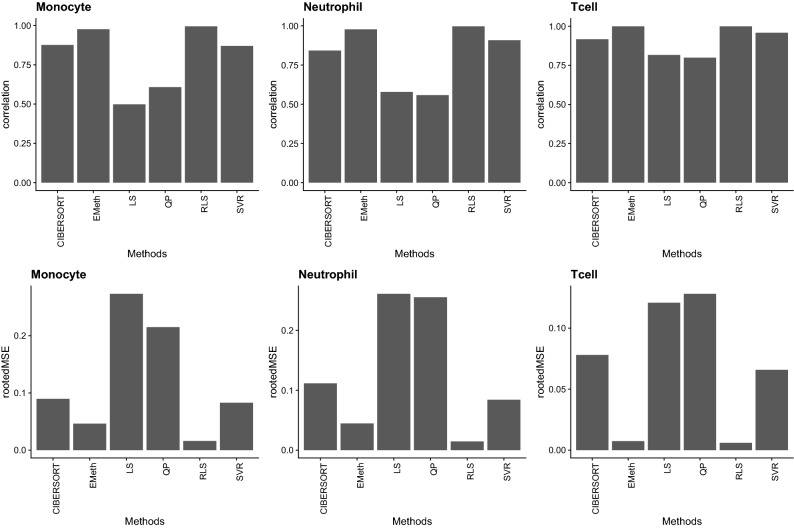
Evaluation of different methods using in silico mixtures. The upper row shows the correlation between the estimated proportions and the true proportions for each cell type and each method. The lower row displays the RMSE. The noise level parameter *c* were set as 5 to better demonstrate the difference across methods, and the conclusions are the same for other values of *c*. These plots were generated using R version 3.6.2^27^.

**Figure 3:**
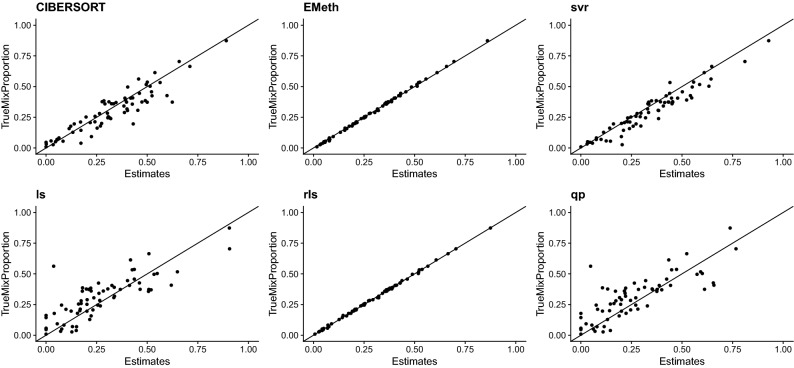
Evaluation of cell type proportion estimates for CD4 T cells using in silico mixtures. The y-axis is the true proportion of CD4 T cells for all 68 samples and the x-axis is the proportion estimates by six different methods. EMeth and RLS give very accurate estimates from methylation data and other methods provide reasonable but less accurate estimates. These plots were generated using R version 3.6.2^27^.

Both EMeth and RLS have accurate estimation results (correlation with true cell type proportions as high as 0.95 and RMSE around $10^{-3}$ for each cell type) and they consistently outperform other methods (Fig. 2). The estimates by EMeth and RLS based on methylation data are more accurate than the estimates by CIBERSORTx based on expression data. The superior performances of EMeth and RLS are more apparent by examining the cell type proportion estimates for each cell type across all the individuals (Fig. 3).

### Results on TCGA data

We studied immune cell type composition for four cancer types (colon cancer, lung adenocarcinoma, lung squamous cell carcinoma, and skin cutaneous melanoma) using the gene expression data (bulk RNA-seq data) and DNA methylation data (Illumina 450k array) from The Cancer Genome Atlas (TCGA). We applied EMeth using the reference data of the seven immune cell types used in our simulation study. To estimate cell type proportions using gene expression data, we applied CIBERSORTx^26^ using its default reference of 22 immune cell types (referred to as LM22), followed by cell size correction. Cell size correction is needed because CIBERSORTx estimates the proportion of gene expression from each cell type, and thus if one cell type has more transcripts per cell, its proportion will be over-estimated^28^. We further collapsed the cell type proportion estimates to the seven cell types used for DNA methylation-based deconvolution to facilitate direct comparisons.

**Figure 4:**
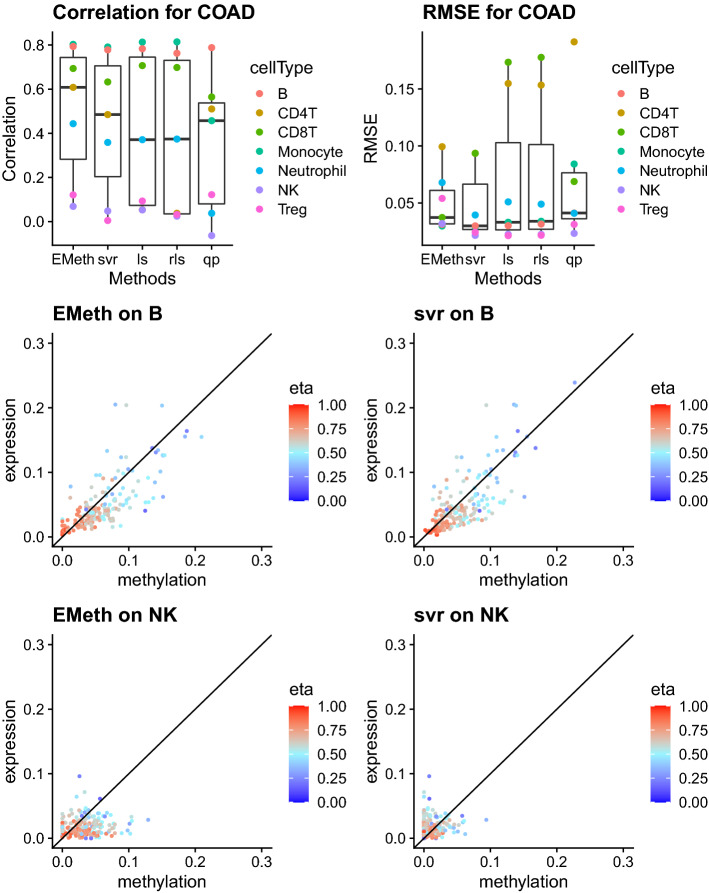
Compare cell type proportion estimates from colon cancer samples The top row illustrates the consistency (in terms of correlation and RMSE) between cell type proportion estimates from methylation data (by multiple methods) and gene expression data (by CIBERSORTx). The middle row displays the estimation result for B cells, an example where cell type proportion estimates from DNA methylation and gene expression are highly consistent. Each point corresponds to one tumor sample and it is colored by tumor purity (eta). The bottom row shows the results for natural killer cell, an example where cell type proportion estimates from DNA methylation and gene expression have very low correlations. These plots were generated using ggplot2 version 3.3.1^24^.

Across all the cancer types, the cell proportion estimates using DNA methylation and gene expression have good consistency for B cells and monocytes, with correlation $> 0.7$ for most cancer types or deconvolution methods (Fig. 4 and Figures S23-S25 in Supplementary Materials). The proportions of CD8T have good consistency by correlation, though the estimates using gene expression is often higher, leading to relatively large RMSE when comparing the estimates using the two types of data (Figures S26-S30). Consistency for CD4T and neutrophil varies according to cancer types or methods. The proportion estimates of natural killer and T regulatory cells (Treg) by gene expression and DNA methylation often have very low correlations (Figures S31-S34), which could be partly due to the low abundance of these two cell types.

Without independent cell type proportion estimates by other approaches for the same samples (e.g., flow cytometry), it is not possible to assess which type of omic data (gene expression or DNA methylation) provide more accurate estimates. We sought to assess this indirectly by examining the associations between cell type proportions and two variables of interest: mutation burden and survival time.

**Figure 5:**
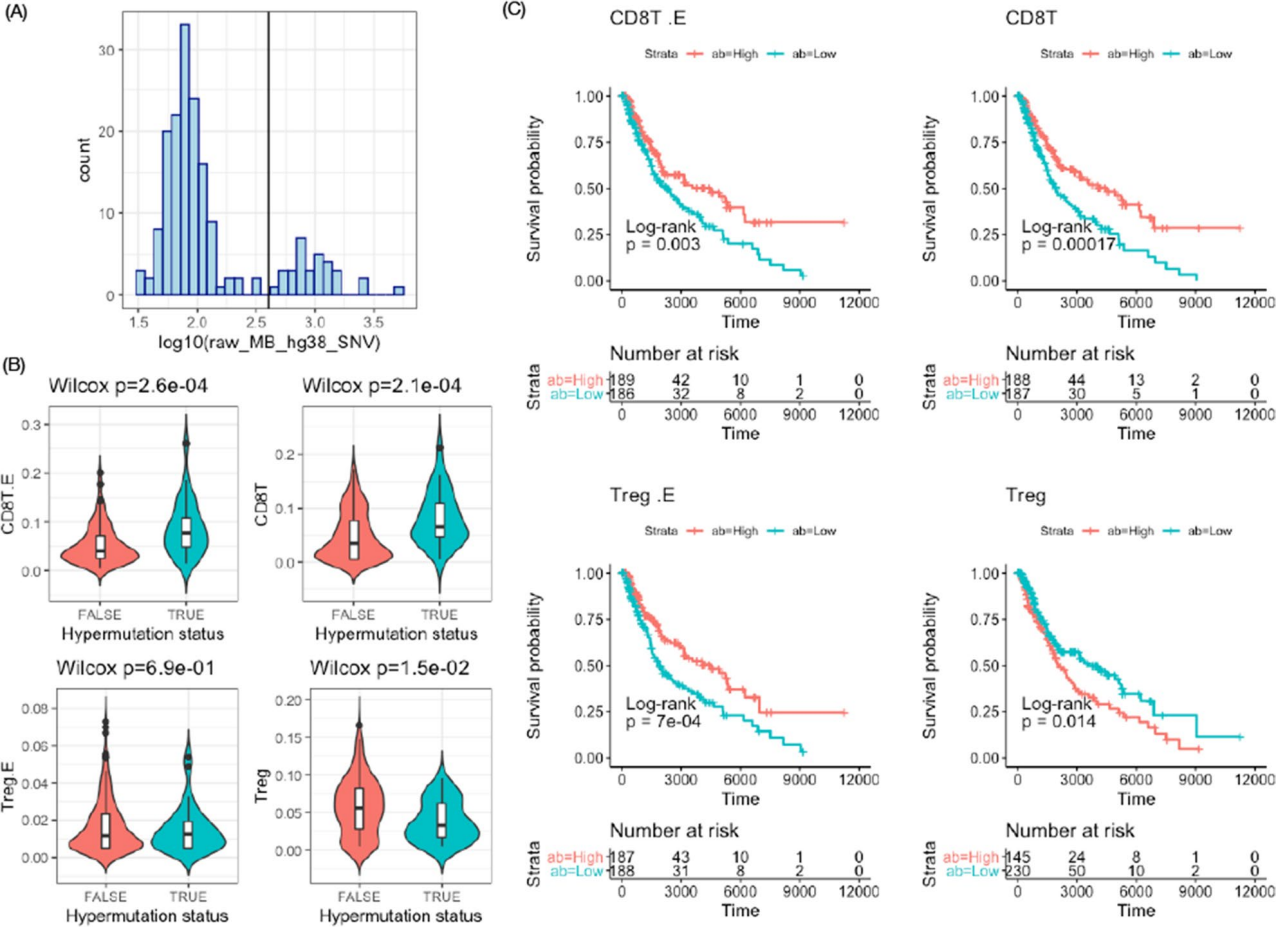
Association analysis for cell type proportions. (**A**) The distribution of mutation loads across colon cancer patients. The vertical line indicates the cutoff to classify samples to be hypermutated. (**B**) Comparison of cell type proportions of the hypermutated samples versus non-hypermutated colon cancer samples. The cell proportions estimated by gene expression are labeled by a suffix “.E”. (**C**) Associations between survival time and cell type proportions for melanoma patients. All the samples are divided into two groups based the median of cell type proportion estimates (abundance/ab high or low). More than half of the Treg proportions (estimated by DNA methylation) are 0’s and thus the group of “ab high” is less than 50% of the samples. These plots were generated using ggplot2 version 3.3.1^24^ and survminer 0.4.8^32^.

We used the mutation burden calculated in an earlier work of our group^29^, where we defined the mutation burden of a tumor sample as the total number of non-silent single nucleotide variants (SNVs) plus the number of indels. A tumor cell with higher mutation burden may present more mutated peptides on its cell surface, and thus has higher chance to be recognized by the immune system. This will in turn triggers immune infiltration in tumor, particularly CD8 T cells. Colon cancer is an ideal case to study the relation between mutation burden and immune infiltration because there is a subset of tumor samples that are hypermutated (Fig. 5A). We observe that CD8T cells proportions (either the ones estimated using gene expression/CIBERSORTx or DNA methylation/EMeth) are indeed higher in the hypermutated subset (Fig. 5B). The Treg proportions estimated by gene expression are similar between the two subsets. In contrast, Treg proportions estimated by DNA methylation are lower in the hypermutated subsets (Fig. 5B), consistent with earlier finding that Tregs are depleted in hypermutated colon cancer samples^30^. Angelova et al.^30^ used gene expression to infer immune cell abundance. Instead of deconvolution based on references, as was done by CIBERSORTx or EMeth, they inferred the abundance of a cell type using weighted average of a group of genes that are highly expressed in that cell type. Therefore the method of Angelova et al. is less quantitative (since they do not give exact proportions) but more robust because they do not fit a deconvolution model. The fact that their conclusions match our EMeth results (i.e., Tregs are depleted in hypermutated colon cancer samples) provides a strong support for the accuracy of EMeth.

We assessed the associations between survival time and cell type proportion estimates (from either gene expression or DNA methylation) in all cancer types and only detected significant associations in melanoma (SKCM) samples, therefore here we focused on the results from SKCM samples. The association patterns for CD8T are consistent between cell type proportions estimated by gene expression and DNA methylation: higher abundance of CD8T is associated with better survival outcomes (see Fig. 5C for log-rank test results and Table S4 in the Supplementary Materials for Cox regression results). This is expected since more immune infiltration leads to better survival outcomes^2,3^. Since T regulatory cells function in suppressing tumor-specific immune responses^31^, negative association between Treg abundance and survival time is expected. This is the case for the DNA-methylation-based Treg proportions, but not true for the gene expression-based Treg proportions (Fig. 5C and Table S4 in Supplementary Materials).

The discrepancy of Treg proportion estimates by gene expression and DNA methylation could be due to inaccuracy of cell type-specific reference. While DNA methylation of bulk tumor samples and reference were both measured by Illumina 450k arrays, the gene expression data were measured by two different platforms: RNA-seq for bulk tumor samples and microarray for reference. Such platform difference could lead to bias in cell proportion estimates. To address this potential issue, we prepared another gene expression reference of 11 cell types using single cell RNA-seq (scRNA-seq) data of 16,291 immune cells from 48 tumor samples of melanoma patients^33^, and we refer to it as SF11 reference matrix. For each cell type, gene expression in this scRNA-seq data has positive, but sometime moderate correlation with the gene expression in the LM22 matrix (Figure S34), reflecting difference due to two platforms and two types of samples (blood samples for LM22 and tumor samples for SF11). We selected 594 genes for the SF11 signature matrix based on the results of differential expression analysis. The expression of a few signature genes were illustrated in Figures S35-S40. See Section 5.2 of the Supplementary Materials for more details on how this SF11 signature was prepared, and the complete pipeline is available at https://github.com/Sun-lab/IT-predictor/tree/master/R/SF2018_analysis.

We estimated cell proportions in the TCGA SKCM samples by CIBERSORTx, using the SF11 signature matrix, and compared the estimates with the estimates based on gene expression LM22 signature and DNA methylation EMeth signature (Figure S41). The cell types that have relatively high correlation between LM22 and EMeth estimates, such as B cells or CD8T cells, also have high correlation between LM22 and SF11 estimates. In contrast, Treg proportion estimates using SF11 and LM22 have weaker correlation. In fact, the Treg proportion estimates using LM22 has stronger correlation with SF11 estimates in 3 other cell types (correlation 0.74, 0.55, and 0.54 for B cells, CD4T, and monocyte, respectively) than with Treg itself (correlation 0.5). The Treg proportion estimates using either SF11 or LM22 have negative correlations with EMeth estimates (correlation -0.18 and -0.2 for SF11 and LM22 estimates, respectively, Figure S41). It is worth noting that for some cell types, the proportion estimates from gene expression and DNA methylation have higher correlations than the estimates from two gene expression signatures. For example, for B cells, the correlations between EMeth estimates and SF11/LM22 estimates are 0.84 and 0.90, respectively, while the correlation between SF11 and LM22 estimates is 0.79.

## Method

### EMeth overview

Suppose we have *I* bulk samples, each one of which consists of a mixture of $Q+1$ cell types. Assume the cell type-specific methylation are known for the first *Q* cell types, and the mixture proportion is denoted by $\rho _{qi}$ for the *q*-th cell type in the *i*-th sample. The $(Q+1)$-th cell type is a special one that does not have cell type-specific methylation information but has known cell type proportions in bulk samples, denoted by $\eta _i$ for sample *i*. In tumor samples, this cell type can be tumor cell and its proportion is tumor purity that can be estimated by copy number data. We also allow aberrant CpGs whose cell type-specific methylation is inconsistent between bulk tissue sample and reference of cell type-specific methylation.

Denote the methylation (in the scale of $\beta$-value) at the *k*-th CpG and the *i*-th bulk sample by $z_{ki}$. We model $z_{ki}$ by a mixture of two normal distributions.

1 $$\begin{aligned} z_{ki} \sim \pi _{ai} {\mathcal {N}}(\mu _{ki},\sigma _{a}^2w_{ki})+\pi _{ci} {\mathcal {N}}(\mu _{ki},\sigma _{c}^2w_{ki}), \end{aligned}$$

Here $\pi _{ai}$ and $\pi _{ci}$ are the probabilities that a CpG being aberrant or consistent in sample *i*, respectively, and $\pi _{ai}+\pi _{ci}=1$. ${\mathcal {N}}(\mu _{ki},\sigma _{a}^2w_{ki})$, the aberrant component, is a normal distribution with mean value $\mu _{ki}$ and variance $\sigma _{a}^2w_{ki}$. The consistent component ${\mathcal {N}}(\mu _{ki},\sigma _{c}^2w_{ki})$ is another normal distribution with the same mean value but smaller variance: $\sigma _c < \sigma _a$. The decomposition into multiple cell types is achieved by a regression model $\mu _{ki}=\eta _i\nu _{0k}+\sum _{q=1}^Q\rho _{qi}\nu _{qk}$, where $\eta _i + \sum _{q=1}^Q\rho _{qi}=1$. Furthermore, we assume that $\eta _i$ is known for each sample and $\nu _{qk}$ is known for each CpG and each cell type. From this model, we can see that the cell type-specific DNA methylation in the special cell type ($\nu _{0k}$) is estimable because it can be considered as the regression coefficient for $\eta _i$ in a CpG-specific regression. As the consequence, a reasonable sample size and sufficient variation of cell type proportions across tissue samples is needed to estimate $\nu _{0k}$.

By default, we set $w_{ki}\equiv 1$, other choices of $w_{ki}$ may further improve the performance of EMeth. As an example, in the Supplementary Materials we compare original version of EMeth with a new version **EMeth-Binom** that sets the weights based on the binomial distribution: $w_{ki}= \sum _q \rho _{qi} \nu _{qk}(1-\nu _{qk})+\eta _i\nu _{0k}(1-\nu _{0k})$. EMeth-Binom can provide more accurate estimates of the model parameters but did not improve the estimates of cell type proportions, and thus we focus on the results of standard EMeth with all the weights being 1. In our simulation studies, we simulated DNA methylation using the mean-variance relation specified by EMeth-Binom.

Since we are interested in estimating $\rho _{qi}$, the model can be reorganized as

2 $$\begin{aligned} z_{ki} = \eta _i\nu _{0k}+\sum _{q=1}^Q\rho _{qi}\nu _{qk}+\varepsilon _{ki}, \end{aligned}$$

where $\varepsilon _{ki} \sim \pi _{ai} {\mathcal {N}}(0,\sigma _{a}^2w_{ki})+\pi _{ci} {\mathcal {N}}(0,\sigma _{c}^2w_{ki})$ and $\sum _{q=1}^Q{\rho }_{qi}=1-\eta _i,\rho _{qi}\ge 0$. For this mixture of regression model, it is natural to propose an EM-type algorithm to maximize the likelihood. The reference data often have strong collinearity between two closely related cell types. We provide a simple remedy to this issue by adding a ridge penalty term: $\sum \rho _{qi}^2$. The missing data in EM algorithm is denoted by $\gamma _{ik}$, which denotes whether the *j*-th CpG is aberrant or consistent for the *i*-th bulk tumor sample. To conclude, in our model the complete penalized likelihood is given by

3 $$\begin{aligned} \ell (\rho )&= \sum _k\sum _i \left[ \gamma _{ki} \log \pi _{ai} + (1-\gamma _{ki})\log (1-\pi _{ai}) \right] \nonumber \\ \nonumber&- \frac{1}{2} \sum _k\sum _i \gamma _{ki} \left( \log \sigma _{a}^2w_{ki} +\frac{e_{ki}^2}{\sigma _{a}^2w_{ki}}\right) \nonumber \\ \nonumber&-\frac{1}{2}\sum _k\sum _i(1-\gamma _{ki})\left( \log \sigma _{c}^2w_{ki} +\frac{e_{ki}^2}{\sigma _{c}^2w_{ki}}\right) \nonumber \\&-\lambda \sum _{i,q}\rho _{qi}^2+Const \; , \end{aligned}$$

where $$e_{ki} = z_{ki}-\eta _i\nu _{0k}-\sum _{q=1}^Q\rho _{qi}\nu _{qk}$$.

The details of the computation of EMeth are presented in Section 1 of the Supplementary Materials. We also consider a variant by replacing the normal distribution with Laplace distribution (**EMeth-Lap**). Note that the Laplace distribution is also in the location-scale family, the main difference is to replace the $\ell _2$-loss corresponding to normal distribution to $\ell _1$-loss corresponding to the Laplace distribution, i.e., replace $e_{ki}^2$ by $|e_{ki}|$. We still include the ridge penalty term in **EMeth-Lap**. Its performance is similar to standard version of EMeth and thus we focus on the results of the standard EMeth in this paper.

## Discussion

Estimates of cell type proportions, particular for immune cell infiltration in tumor samples, are highly valuable for down-stream association analyses. Many efforts have been devoted to this topic and most of the existing works focus on deconvolution of cell type proportions using gene expression data. DNA methylation is an attractive alternative because it is often a more stable than gene expression. Our method, EMeth, is designed for deconvolution using DNA methylation and it has two novelties. One is to allow a subset of aberrant CpGs and thus increase the robustness of the method. The other one is to allow one cell type without reference and estimate the DNA methylation of this cell type. We have demonstrated the advantages of EMeth in simulations studies. In our case study using TCGA data, the proportion of T regulatory cells (Tregs) estimated from gene expression and DNA methylation have very low correlation, and discrepant association results. We found the association results using Treg proportion estimates from DNA methylation is more consistent with the functional roles of Tregs. However, for the cell types with low proportions across most of the samples, e.g., Tregs, the estimation noise may have large impact on the association signals and further studies are warranted.

In this study, we focus on Illumina 450k methylation array because it is the platform where we can find good amount of cell type-specific and bulk tissue DNA methylation data. Since we only select around 900 CpGs for the final deconvolution, our approach should be applicable to the platforms with fewer CpGs such as the Illumina 27k methylation array that still have enough CpGs to make selection. Our method is also applicable to the newer arrays such as the MethylationEPIC arrays with 850k CpGs or whole genome bisulfite sequencing. It is ideal to generate cell type-specific reference using the same platform though we expect the reference generated from 450k array can be used for deconvolution of methylation data from other array platforms, after a new round of CpG selection to match the array platforms. Whether such array based reference is appropriate for deconvolution using bisulfite sequencing data warrants further studies.

Although our EMeth method is motivated by cancer studies (e.g., the special cell type with known proportion is designed to mimic tumor cells), it can be applied to other tissues, such as blood, to study the cell type composition associated with other disorders such as autoimmune diseases. Our simulation study is designed to mimic the level of noise observed in cancer studies (Table 1). Since the noise in tumor tissues is often larger than other tissues, the simulation study could be considered as a more challenging case. In contrast, our in silico study used a very unique and valuable dataset with cell type-specific DNA methylation measured in $\sim 200$ individuals. The unique advantage of this dataset is that we can generate mixture of multiple cell types while preserving the biological variation across individuals. At the same time, we can use a subset of the individuals to estimate cell type-specific reference, and evaluate deconvolution performance using the remaining individuals, and thus preserve the biological variation between reference data and bulk tissue samples. Because the reference and bulk are generated from the same study and thus we can avoid batch effects, this in silico study represents an easier case. The fact that EMeth performs well in both cases suggest it has robust performance in different settings.

A limitation for DNA methylation-based deconvolution, compared with gene expression-based ones is the lack of cell type-specific DNA methylation data in different cell types. For example, in our deconvolution analysis, we only considered seven cell types. Some of the cell types could be further refined and it is desirable to include more cell types. We believe as more works demonstrate the value of cell type-specific DNA methylation data, and with the technique advancement, more of such reference data will be generated in the near future. Although EMeth allows a special cell type without reference, we do assume the proportions of this cell type are known. EMeth cannot handle an arbitrary missing cell type without either cell type-specific reference or cell type proportions.

When the cell type-specific reference is inappropriate, EMeth may find the vast majority of of the CpG probes are aberrant. With a very limited number of CpG probes usable, there is risk of over-fitting. EMeth will issue a warning in such situation and suggest improvement of the cell type-specific references.

Single cell DNA methylation data is potential resource to generate cell type-specific reference. However, currently such data are often very sparse and expensive to generate. A promising direction is to leverage cell type-specific scRNA-Seq data to generate corresponding cell-type-specific DNA methylation reference matrix^34^. In the near future, the technique development may be more mature to measure both gene expression and DNA methylation in the same single cell^35^, and thus making it feasible to combine gene expression and DNA methylation to infer cell type-specific DNA methylation reference matrix.

Finally, we conclude with the computational efficiency of EMeth. The EM is known to be slow in convergence. Though in our application, we use EM for a classical mixture model where convergence is relatively fast. For example, in our analysis of TCGA melanoma data from 393 bulk tumor samples, it took about 10 minutes to run the EM algorithm 20 times (4 penalty parameters and 5-fold cross validation), using a MacBook Pro with 2.3GHz CPU and single thread. The pipelines for analyzing simulated data and TCGA data can be found at https://github.com/Sun-lab/dMeth.

## Supplementary Information

Supplementary Information
