# Supplementary material for: EMeth: An EM algorithm for cell type decomposition based on DNA methylation data
Source: Sci Rep. 2021 Mar 11;11:5717. doi: 10.1038/s41598-021-84864-9 (PMC7952399; doi:10.1038/s41598-021-84864-9)
Supplement: Supplementary file 1 — Supplementary Information [file 41598_2021_84864_MOESM1_ESM.pdf]

Supplementary Materials for "EMeth: An EM algorithm for cell type decomposition based on DNA methylation data"

Hanyu Zhang[1], Ruoyi Cai[2], James Dai[3], and Wei Sun[2,3,4*]
[1]Department of Statistics, University of Washington
[2]Department of Biostatistics, University of Washington
[3]Public Health Science Division, Fred Hutchinson Cancer Research Center
[4]Department of Biostatistics, University of North Carolina

January 3, 2021

Contents

1 Algorithm Implementation Details **2**
1.1 EMeth 2
1.2 Benchmark Algorithms 3

2 Preparation of Cell Type-specific DNA methylation **4**

3 Simulation study **6**
3.1 Study Details 6
3.2 Additional Simulation Results 7
3.2.1 Parameter Estimates 7
3.2.2 Estimation Error under Different Settings 9
3.3 Additional Simulation Results with larger proportion of aberrant CpG probes 18

4 BLUEPRINT Data **19**

5 TCGA Data **19**
5.1 Additional results using LM22 gene expression reference 19
5.2 Additional results using SF11 gene expression reference 32

*wsun@fredhutch.org

1 Algorithm Implementation Details

1.1 EMeth

Recall that our model for the methylation (β-value) at the k-th CpG and the i-th sample, denoted by z_{ki}, is given by a mixture of two normal distributions

$$z_{ki} \sim \pi_{ai}\mathcal{N}(\mu_{ki}, \sigma_a^2 w_{ki}) + \pi_{ci}\mathcal{N}(\mu_{ki}, \sigma_c^2 w_{ki}),$$

where the components a and c correspond to "aberrant" CpG and "consistent" CpG, respectively. We assume there are $Q+1$ cell type. For Q types, we have reference of cell type-specific gene expression (ν_{qk}'s) and seek to estimate their proportions, denoted by ρ_{qi} for $1 \le q \le Q$. For one cell type, we know its proportion (denoted by η_i) and seek to estimate it cell type-specific expression (ν_{0k}). Then z_{ki} can be written as

$$z_{ki} = \mu_{ki} + \varepsilon = \eta_i \nu_{0k} + \sum_{q=1}^{Q} \rho_{qi}\nu_{qk} + \varepsilon_{ki}, \tag{1}$$

with ε_{ki} follows a normal mixture distribution, and the cell type proportions satisfy that $\sum_{q=1}^{Q} \rho_{qi} = 1 - \eta_i, \rho_{qi} \ge 0$.

We introduce missing data y_{ki} to denote whether the data z_{ki} belongs to mixture component a or c. Then the complete log likelihood of data (z_{ki}, y_{ki}) is

$$l(z_{ki}, y_{ki} | \{\rho_{qi}\}, \nu_{0k}, \sigma_a, \sigma_c) = y_{ki} \log\left[\pi_{ai}\mathcal{N}(z_{ki}; \mu_{ki}, \sigma_a^2 w_{ki})\right] + (1 - y_{ki}) \log\left[\pi_{ci}\mathcal{N}(z_{ki}; \mu_{ki}, \sigma_c^2 w_{ki})\right].$$

Next we describe the E-step and the M-step of the EM algorithm.

E step. In this step we take expectation of the complete log likelihood given parameters $\rho_{qi}, \nu_{0k}, w_{ki}, \sigma_a^2, \sigma_c^2$. As for a standard EM algorithm for a mixture model, this requires the estimation of

$$\begin{aligned}\gamma_{ki} = \mathbb{E}[y_{ki} | z_{ki}, \mu_{ki}, \{\rho_{qi}\}, w_{ki}, \sigma_a^2, \sigma_c^2] &= Pr\left(y_{ki} = 1 | z_{ki}, \mu_{ki}, \{\rho_{qi}\}, w_{ki}, \sigma_a^2, \sigma_c^2\right) \\ &= \frac{\pi_{ai}\mathcal{N}(z_{ki}; \mu_{ki}, \sigma_a^2 w_{ki})}{\pi_{ai}\mathcal{N}(z_{ki}; \mu_{ki}, \sigma_a^2 w_{ki}) + \pi_{ci}\mathcal{N}(z_{ki}; \mu_{ki}, \sigma_c^2 w_{ki})}.\end{aligned}$$

We denote the collection of parameters as $\Theta_{ki} = (z_{ki}, \mu_{ki}, \{\rho_{qi}\}, w_{ki}, \sigma_a^2, \sigma_c^2)$.

M step For one sample and one probe, we aim to increase the following expected log likelihood (i.e., the Q function)

$$Q(\Theta_{ki}; \Theta_{ki}^{old}) = \gamma_{ki} \log\left[\pi_{ai}\mathcal{N}(z_{ki}; \mu_{ki}, \sigma_a^2 w_{ki})\right] + (1 - \gamma_{ki}) \log\left[\pi_{ci}\mathcal{N}(z_{ki}; \mu_{ki}, \sigma_c^2 w_{ki})\right].$$

Now we aggregate all the CpGs and samples and add a ridge penalty. In M step we find MLE of

$$\begin{aligned}Q(\mathbf{\Theta}; \mathbf{\Theta}^{old}) &= \sum_k \sum_i \gamma_{ki} \log\left[\pi_{ai}\mathcal{N}(z_{ki}; \mu_{ki}, \sigma_a^2 w_{ki})\right] + (1 - \gamma_{ki}) \log\left[\pi_{ci}\mathcal{N}(z_{ki}; \mu_{ki}, \sigma_c^2 w_{ki})\right] \\ &= \sum_k \sum_i \left(\gamma_{ki} \log \pi_{ai} + (1 - \gamma_{ki}) \log(1 - \pi_{ai})\right) + Const - \lambda \sum_i \sum_q \rho_{qi}^2 \\ &\quad - \frac{1}{2} \sum_k \sum_i \gamma_{ki} \left(\log \sigma_a^2 w_{ki} + \frac{(z_{ki} - \eta_i \nu_{0k} - \sum_{q=1}^{Q} \rho_{qi}\nu_{qk})^2}{\sigma_a^2 w_{ki}} \right) \\ &\quad - \frac{1}{2} \sum_k \sum_i (1 - \gamma_{ki}) \left(\log \sigma_c^2 w_{ki} + \frac{(z_{ki} - \eta_i \nu_{0k} - \sum_{q=1}^{Q} \rho_{qi}\nu_{qk})^2}{\sigma_c^2 w_{ki}} \right).\end{aligned}$$

Considering the number of parameters, we optimize them separately. For $\pi_{ai}, \pi_{ci} = 1 - \pi_{ai}$ it is direct to update

$$\pi_{ai} = \frac{\sum_k \gamma_{ki}}{K}, \tag{2}$$

where K is the total number of CpG probes.

Now we turn to the estimation of $\rho_{qi}, \nu_{0k}, \sigma_a^2 w_{ki}, \sigma_c^2 w_{ki}$. The solution to maximize the expected log likelihood is not analytical. Therefore we first fix $\sigma_a^2 w_{ki}, \sigma_c^2 w_{ki}$ then the maximum likelihood solution of ν_{0k} and ρ_{qi} is given by minimizing

$$\sum_k \sum_i (\frac{\gamma_{ki}}{\sigma_a^2 w_{ki}} + \frac{1-\gamma_{ki}}{\sigma_c^2 w_{ki}}) \left(z_{ki} - \eta_i \nu_{0k} - \sum_{q=1}^{Q} \rho_{qi} \nu_{qk} \right)^2 + \lambda \sum_i \sum_q \rho_{qi}^2$$

with some constraints. Since ν_{0k} remain the same across the samples and ρ_{qi} is same for different CpGs, we will iteratively estimate these two parameters. First a fixed ρ_{qi} we can optimize ν_{0k} across samples by weighted least squares. Then from this fixed ν_{0k} we further decrease the loss function by a constrained quadratic programming to get ρ_{qi}.

With fixed ρ_{qi}, ν_{0k} the MLE of σ_a^2, σ_c^2 can be computed by

$$\sigma_a^2 = \frac{\sum_k \sum_i \gamma_{ki}(z_{ki} - \eta_i \nu_{0k} - \sum_{q=1}^{Q} \rho_{qi}\nu_{kq})^2 / w_{ki}}{\sum_k \sum_i \gamma_{ki}} \quad (3)$$

$$\sigma_c^2 = \frac{\sum_k \sum_i (1-\gamma_{ki})(z_{ki} - \eta_i \nu_{0k} - \sum_{q=1}^{Q} \rho_{qi}\nu_{kq})^2 / w_{ki}}{\sum_k \sum_i (1-\gamma_{ki})} \quad (4)$$

To summarize, the iterations in our (Generalized) EMeth algorithm is repeating the following procedures until convergence.

1. Update π_{ai}, π_{ci} according to (2).
2. Update ν_{0k} by weighted least squares.
3. Plug in the updated ν_{0k}, then update ρ_{qi} with constraints by quadratic programming.
4. Update σ_a^2, σ_c^2 from (4).
5. Update γ_{ki}

We generate random numbers to initialize all parameters except for ρ. The initialization of ρ is given by estimation from non-negative least squares and then truncate to have a total $1 - \eta_i$ for each sample. Then ν_0 is initialized by one step of estimation given the initialized estimates of proportions. The variances are initialized by a rough estimation of variance across all probes and cell types. For LaplaceEM, the only different is all ℓ_2 loss are replaced by ℓ_1 loss. To solve the minimize absolute value problem, we directly use the optim function provided in R. All these source codes can be found in the GitHub repository EMeth.

1.2 Benchmark Algorithms

We have included for benchmark algorithms for cell type deconvolution.

- lr: Ordinary linear regression implemented by R.
- svr: Support vector regression, implemented by svm function in e1071 package with a linear kernel. C parameter is determined by a fivefold cross-validation in $10^{(0,0.5,1,1.5,2)}$.
- rls: Robust linear regression, implemented by rlm function in MASS package. We take a huber loss.
- qp: Quadratic programming, implemented by quadprog package.

For lr,svr,rls, the coefficients are first fitted without any constraint. Then the negative coefficients are forced to be zero and then all coefficients for one sample are renormalized to have sum one. All four benchmark algorithms perform cell type deconvolution sample by sample.

2 Preparation of Cell Type-specific DNA methylation

To collect cell type-specific DNA methylation on immune cells, we searched for the DNA methylation data of individual immune cell types in the European Nucleotide Archive (ENA). Then we manually inspected each dataset to exclude the cases in case-control comparison or to exclude study that appear to have very strong batch effects. Finally we included Illumina 450k array methylation data for 189 samples, from six studies [1–6] and seven cell types (Table S1).

Table S1: Sample size for each study and each cell type

	B	CD4T	CD8T	Monocyte	Neutrophil	NK	Treg
Coit	0	0	0	0	15	0	0
Limbach	0	31	31	0	0	0	0
Reinius	6	6	6	6	6	6	0
Reynolds	0	23	0	34	0	0	0
Schlums	0	0	0	0	0	15	0
Zhang	0	0	0	0	0	0	4

One challenge to use this dataset is that most studies (with the exception Reinius et al. [1]) only have methylation data from one or two cell types and thus created there are confounding between batch effects and cell types. To overcome this problem, we first apply quantile normalization (using R function `normalize.quantiles` from package `preprocessCore` [7]) to normalize the data of each cell type from Reinius et al. [1]. Then we aligned all the other datasets to Reinius et al. by setting their quantiles to the quantiles of the corresponding cell type in Reinius et al. Since Reinius et al. does not have the Treg cell type, we set its quantiles to be the same as CD4T quantiles. Then through PCA analysis, we observe that there are still some batch effects, mainly captured by the PC2 (Figure S3-S4).

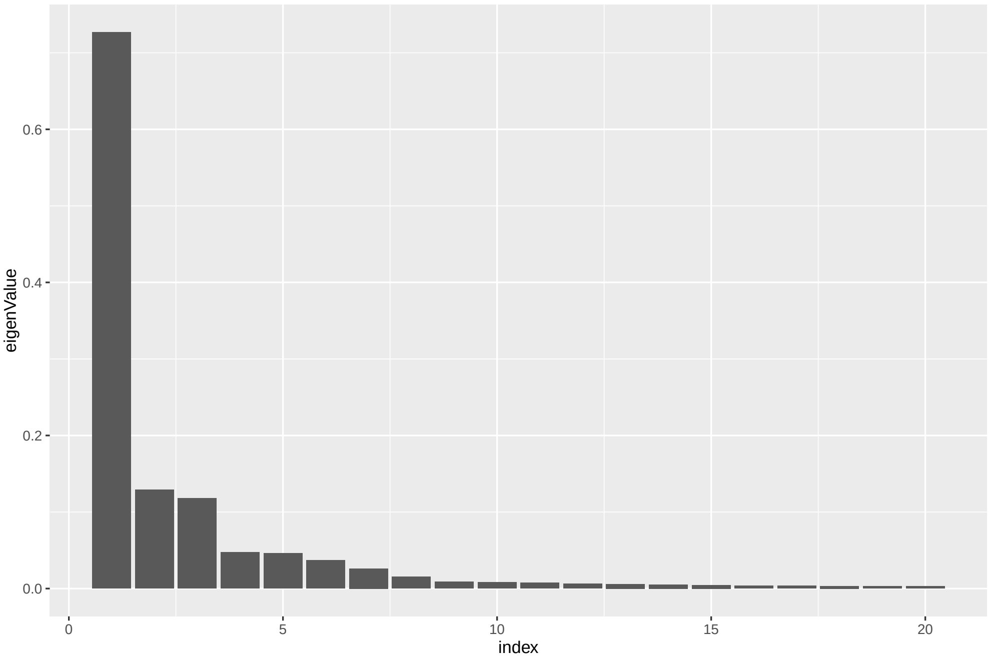

Figure S1: Eigen values for the PCA analysis of the DNA methylation data after quantile normalization.

After regressing out the PC2, the samples with the same cell types are clustered together in the PC

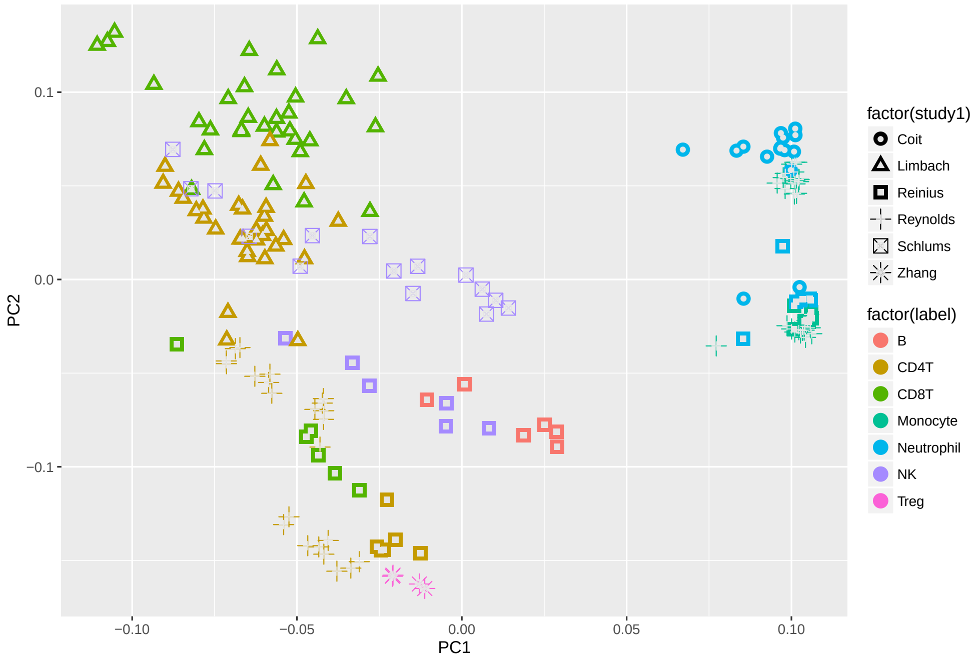

Figure S2: Scatter plot of PC1 vs. PC2 for the DNA methylation data after quantile normalization. Colors indicate cell type and shapes indicate studies.

plot, and we used this version of the DNA methylation data (220,886 CpG probes in 189 samples) in the deconvolution analysis. See `https://github.com/Sun-lab/dMeth` for details.

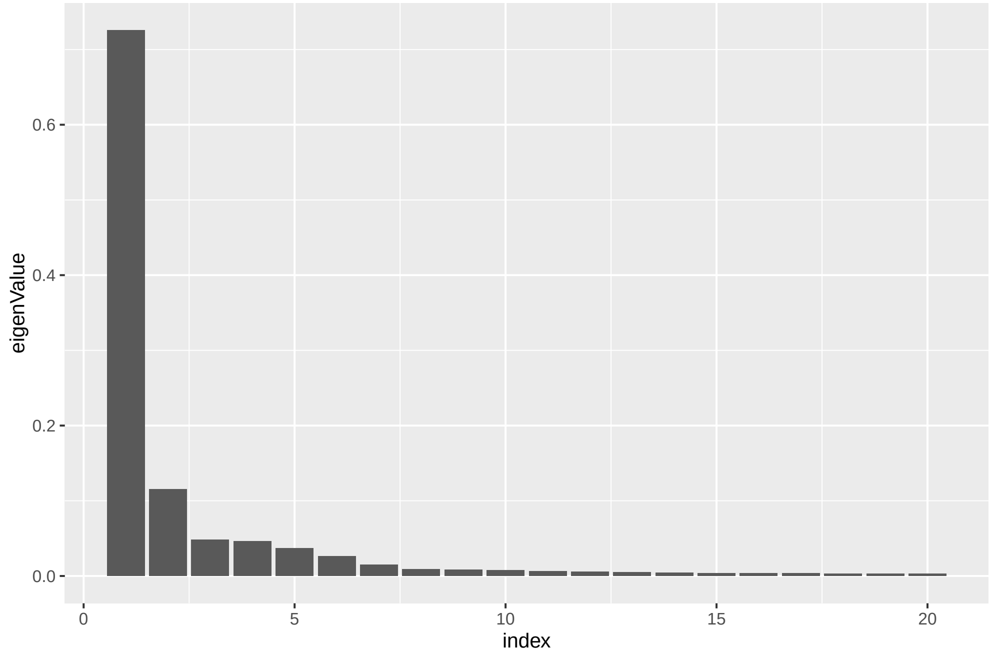

Figure S3: Eigen values for the PCA analysis of the DNA methylation data after quantile normalization and regressing out PC2 from earlier PCA.

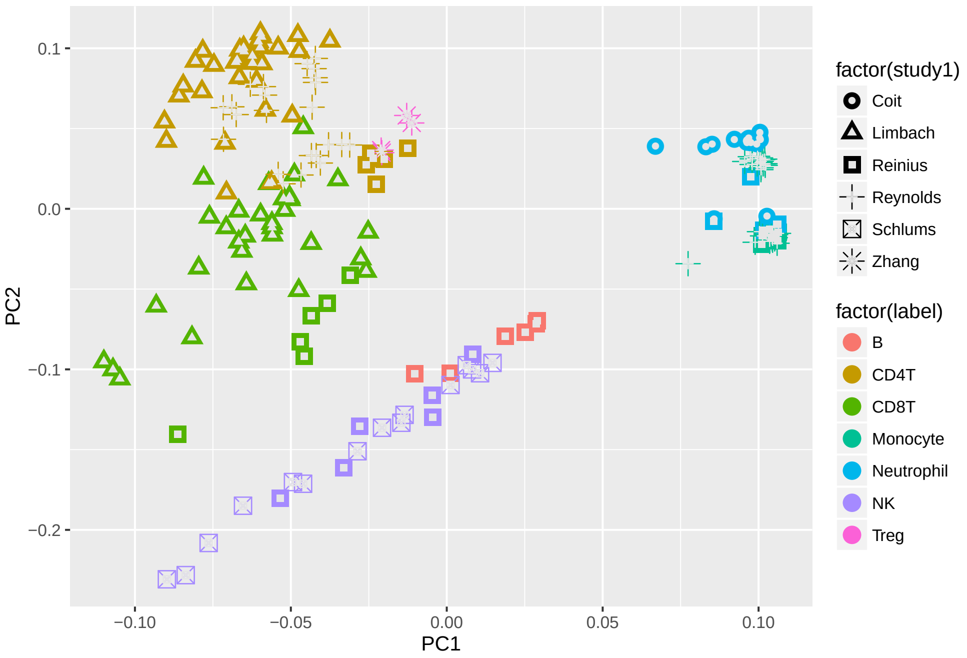

Figure S4: Scatter plot of PC1 vs. PC2 for the DNA methylation data after quantile normalization and regressing out PC2 from earlier PCA. Colors indicate cell type and shapes indicate studies.

3 Simulation study

3.1 Study Details

We randomly divided the 189 samples of cell type-specific DNA methylation data to two subsets approximately evenly. Then we used one subset to generate reference of cell type-specific methylation data, and used the other subset generate the pseudo-bulk samples. We denote these two data sets as $S^{\texttt{ref}}$ and $S^{\texttt{gen}}$, respectively.

To construct reference, we used $S^{\texttt{ref}}$ to select CpGs that are differentially methylated across cell types. The CpGs were selected through 14 comparisons. Seven of them are one cell type versus all the other cell types, and the other seven are based on the lineage relations or DNA methylation similarities: lymphoid vs. myeloid, B cell vs. NK, B cell + NK v. all T cells, CD4T + Tregs vs. CD8T, CD4T vs. Tregs, Neutrophil vs Monocyte, and CD4T vs CD8T. Except for the comparison of CD4T vs Tregs, where no differentially methylated CpG was discovered, we found more than 1,000 differentially methylated CpGs (p-value 0.01 by two sample t-test), and we chose 100 out of them to construct the reference. This step selects a total of 946 non-redundant CpGs. After selecting the probes, we estimate the mean and standard deviation of $S^{\texttt{ref}}$ and $S^{\texttt{gen}}$ for each probe separately, denoted by $\mu^{\texttt{ref}}$ and $\mu^{\texttt{gen}}$. $\mu^{\texttt{ref}}$ will be used as our reference data.

We used $\mu^{\texttt{gen}}$'s to generate pseudo-bulk samples that are mixture of these seven cell types. We select approximately 10% of CpGs as aberrant ones, and replaced their expected methylation values in $\mu^{\texttt{gen}}$ by random samples from a uniform distribution $(0, 1)$. Then we generated the mixture methylation by the following steps.

1. Set $I = 100$, generate $\pi_{ai} \sim Uniform(0.05, 0.15)$.

2. Generate ν_{0k} from Uniform(0,1) for each k. Select σ_c^2 and $\lambda \in (2, 10, 100)$.

3. For each sample i, generate η_i from Unif(0,1). Then generate ρ_{qi} in the following way: first generate seven random variables uniformly distributed on $((1/Q) - 0.1, (1/Q) + 0.1)$, then renormalize ρ_{qi} such that $\sum_{q=1}^{Q} \rho_{qi} = 1 - \eta_i$ for each i. In this study, the number of cell types $Q = 7$.
4. Compute μ_{ki} for sample i and probe k according to the mixture model (equation (1)). Compute the variance by EMeth-Binom weights (i.e., the using binomial distribution assumption: $\mu_{ki}(1 - \mu_{ki})$) and generate normal distribution random variables as z_{ki}.

3.2 Additional Simulation Results

3.2.1 Parameter Estimates

The results in the main text focus on the estimation accuracy of cell type fractions. Here we provide the summary for other parameter estimates. One quantity that is of particular importance is the estimation of the DNA methylation in the special cell type without reference. EMeth provides reasonable accurate estimates. The correlation with true values are around 0.7 in most cases (Table S2). Note that the data were simulated based on EMeth-Binom model, where the variance for the the k-th CpG and i-th sample is $\sigma_c^2 w_{ki}$ or $\sigma_c^2 w_{ki}$ for consistent and aberrant CpGs respectively, where $w_{ki} = \mu_{ki}(1 - \mu_{ki})$. However, when we estimate σ_c^2 or σ_a^2 using EMeth, we only estimate one value for all the CpGs. Therefore, the evaluation of the accuracy of σ_c^2 or σ_a^2 estimates are at best an approximation where we compare our estimates with the mean values of $\sigma_c^2 w_{ki}$ or $\sigma_c^2 w_{ki}$ across all the 946 CpGs.

Table S2: **Other parameters estimated from EMeth in combined data simulation study.** We report the average bias of π for all samples. The correlation between $\widehat{\nu}_0$ and ν_0 (the DNA methylation in the cell type without reference data), and the estimation of σ_a^2, σ_c^2. For each sample, average variance σ_a^2, σ_c^2 of each probe is computed and used to compute the bias in the original case of EMeth. In binomial case, direct estimation of σ_a^2, σ_c^2 is implemented and the bias is with respect to the difference between the estimator and the true value of σ_a^2, σ_c^2

σ_c^2	λ	Methods	median($\widehat{\overline{\pi}} - \overline{\pi}$)	Corr($\widehat{\nu_0}, \nu_0$)	median($\widehat{\sigma_a^2 - \sigma_a^2}$)	median ($\widehat{\sigma_c^2 - \sigma_c^2}$)
0.004	2	EMeth	0.260(0.007)	0.726(0.022)	0.168(0.007)	0.021(0.000)
		EMeth-Binom	0.122(0.005)	0.696(0.032)	0.163(0.012)	-0.002(0.000)
	10	EMeth	0.290(0.008)	0.724(0.022)	0.137(0.006)	0.021(0.000)
		EMeth-Binom	0.140(0.006)	0.693(0.032)	0.131(0.011)	-0.002(0.000)
	100	EMeth	0.324(0.006)	0.725(0.022)	-0.208(0.006)	0.020(0.000)
		EMeth-Binom	0.176(0.004)	0.697(0.032)	-0.214(0.011)	-0.002(0.000)
0.00298(LUSC)	2	EMeth	0.239(0.007)	0.732(0.021)	0.167(0.007)	0.019(0.000)
		EMeth-Binom	0.107(0.005)	0.704(0.031)	0.164(0.013)	-0.001(0.000)
	10	EMeth	0.267(0.008)	0.730(0.022)	0.144(0.007)	0.020(0.000)
		EMeth-Binom	0.123(0.005)	0.701(0.031)	0.140(0.012)	-0.001(0.000)
	100	EMeth	0.304(0.006)	0.731(0.022)	-0.110(0.006)	0.019(0.000)
		EMeth-Binom	0.160(0.004)	0.703(0.031)	-0.116(0.011)	-0.001(0.000)
0.00221(LUAD)	2	EMeth	0.220(0.007)	0.737(0.021)	0.167(0.008)	0.018(0.000)
		EMeth-Binom	0.093(0.005)	0.710(0.030)	0.165(0.015)	-0.001(0.000)
	10	EMeth	0.245(0.008)	0.735(0.021)	0.149(0.007)	0.018(0.000)
		EMeth-Binom	0.108(0.004)	0.707(0.030)	0.147(0.012)	-0.000(0.000)
	100	EMeth	0.284(0.006)	0.735(0.021)	-0.038(0.006)	0.017(0.000)
		EMeth-Binom	0.145(0.005)	0.709(0.030)	-0.043(0.012)	-0.000(0.000)
0.00141(SKCM)	2	EMeth	0.192(0.007)	0.743(0.021)	0.166(0.009)	0.016(0.000)
		EMeth-Binom	0.075(0.003)	0.719(0.028)	0.167(0.017)	-0.000(0.000)
	10	EMeth	0.216(0.008)	0.742(0.021)	0.154(0.008)	0.016(0.000)
		EMeth-Binom	0.087(0.003)	0.716(0.028)	0.154(0.014)	0.000(0.000)
	100	EMeth	0.257(0.005)	0.742(0.021)	0.037(0.006)	0.015(0.000)
		EMeth-Binom	0.122(0.005)	0.716(0.028)	0.033(0.013)	0.000(0.000)
0.00118(COAD)	2	EMeth	0.183(0.007)	0.744(0.020)	0.166(0.009)	0.015(0.000)
		EMeth-Binom	0.069(0.003)	0.721(0.027)	0.168(0.019)	0.000(0.000)
	10	EMeth	0.205(0.008)	0.743(0.021)	0.155(0.009)	0.015(0.000)
		EMeth-Binom	0.080(0.003)	0.718(0.028)	0.156(0.015)	0.000(0.000)
	100	EMeth	0.247(0.005)	0.744(0.021)	0.058(0.007)	0.014(0.000)
		EMeth-Binom	0.114(0.004)	0.719(0.027)	0.054(0.012)	0.000(0.000)
0.001	2	EMeth	0.174(0.007)	0.746(0.020)	0.166(0.009)	0.014(0.000)
		EMeth-Binom	0.063(0.003)	0.724(0.027)	0.168(0.020)	0.000(0.000)
	10	EMeth	0.195(0.007)	0.745(0.020)	0.157(0.008)	0.014(0.000)
		EMeth-Binom	0.074(0.003)	0.721(0.027)	0.158(0.017)	0.000(0.000)
	100	Emeth	0.238(0.005)	0.745(0.020)	0.074(0.007)	0.014(0.000)
		Emeth-Binom	0.106(0.004)	0.721(0.027)	0.071(0.013)	0.000(0.000)

3.2.2 Estimation Error under Different Settings

Next we illustrate the estimation errors of all methods (including EMeth-Binom) under all simulation settings. EMeth and EMeth-Binom perform better than all the other methods for most cell types in all the simulation settings and EMeth perform slightly better than EMeth-Binom in some settings.

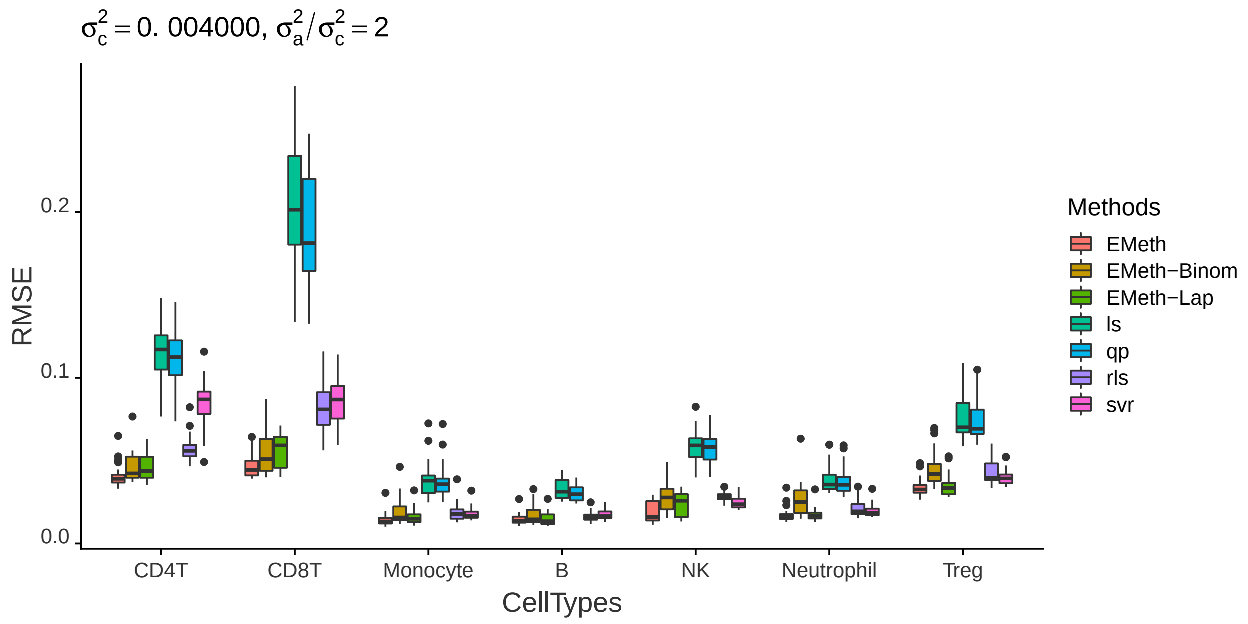

Figure S5: $\sigma^2 = 0.004, \lambda = 2$

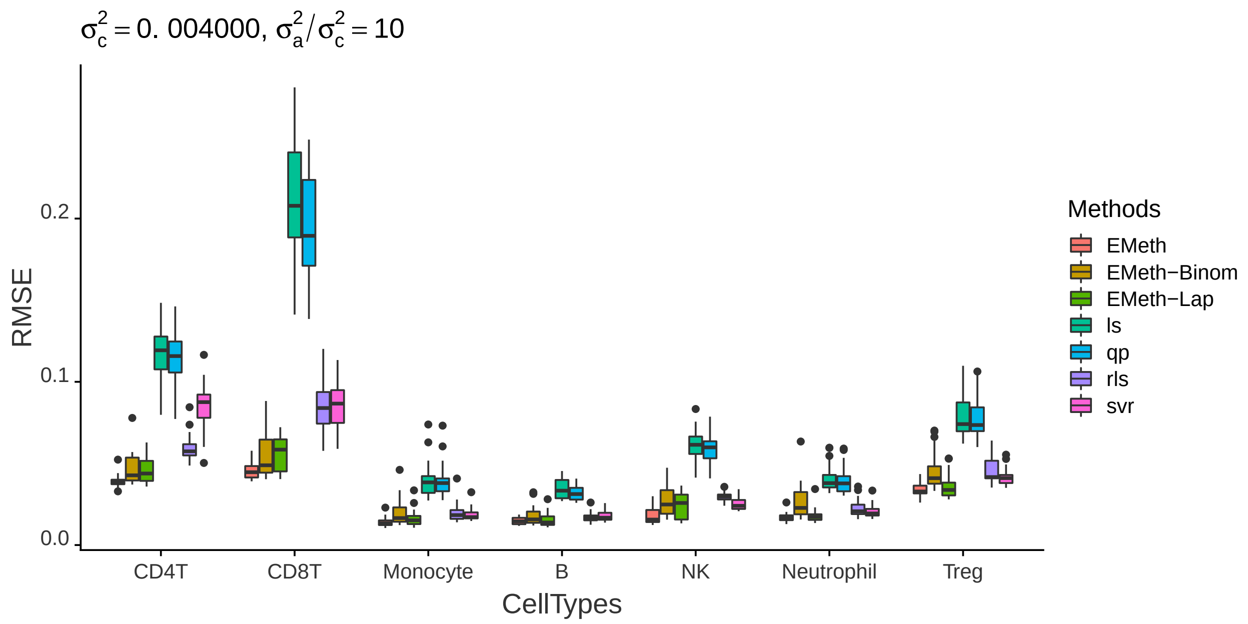

Figure S6: $\sigma^2 = 0.004, \lambda = 10$

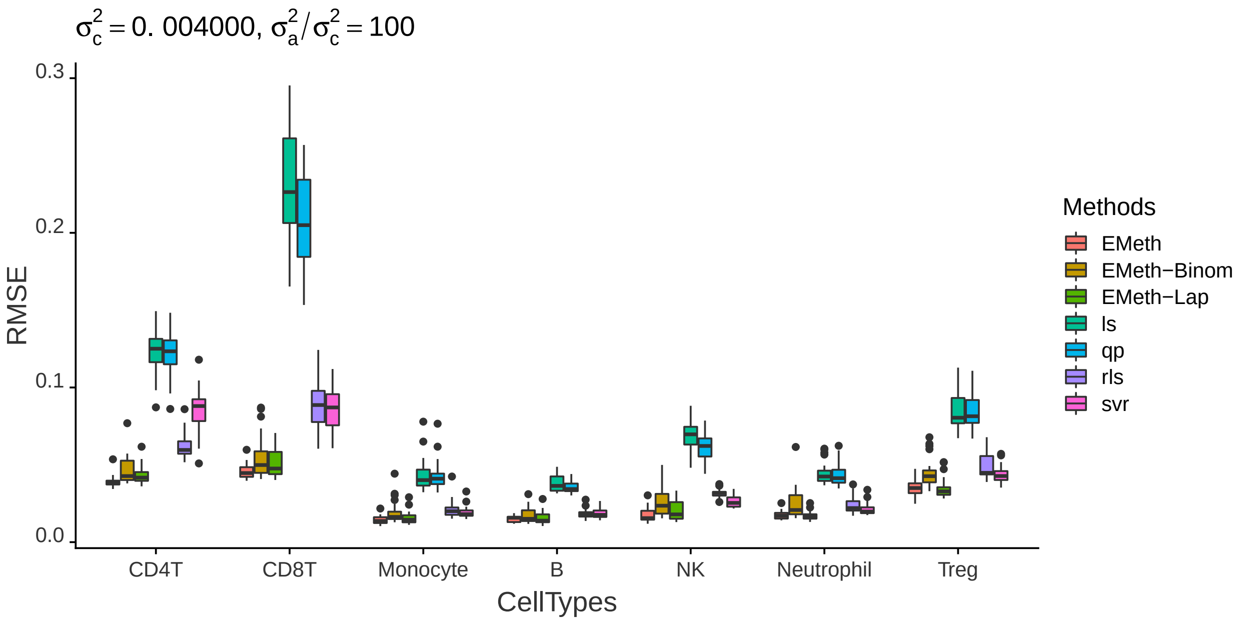

Figure S7: $\sigma^2 = 0.04, \lambda = 100$

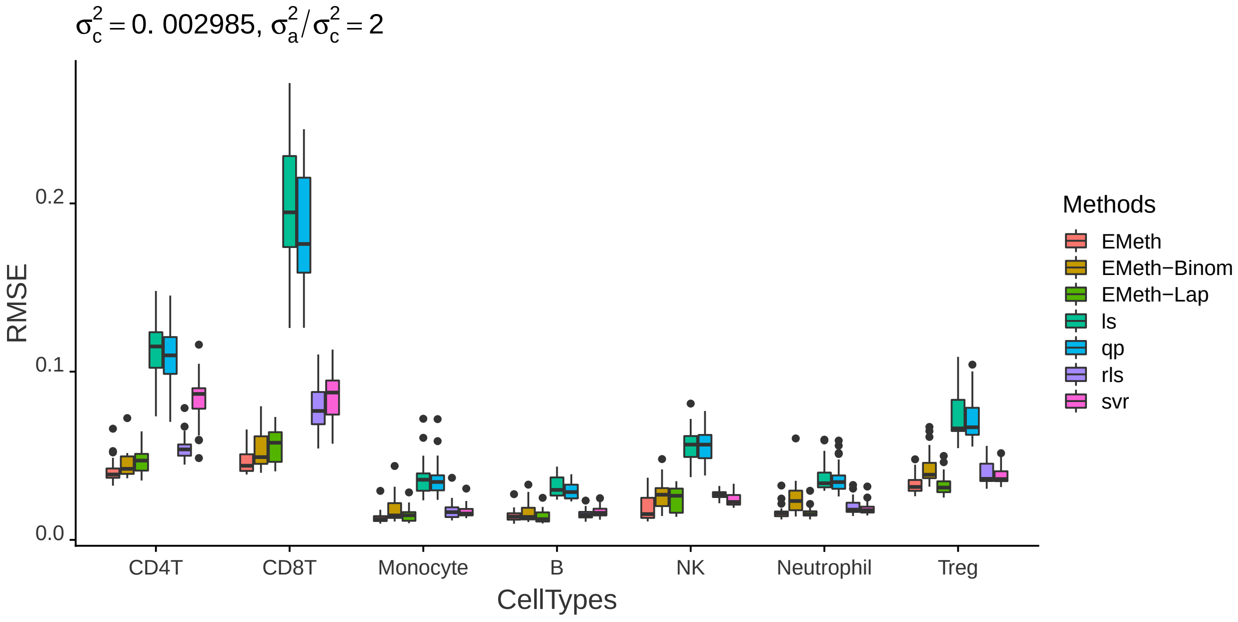

Figure S8: $\sigma^2 = 0.00298, \lambda = 2$

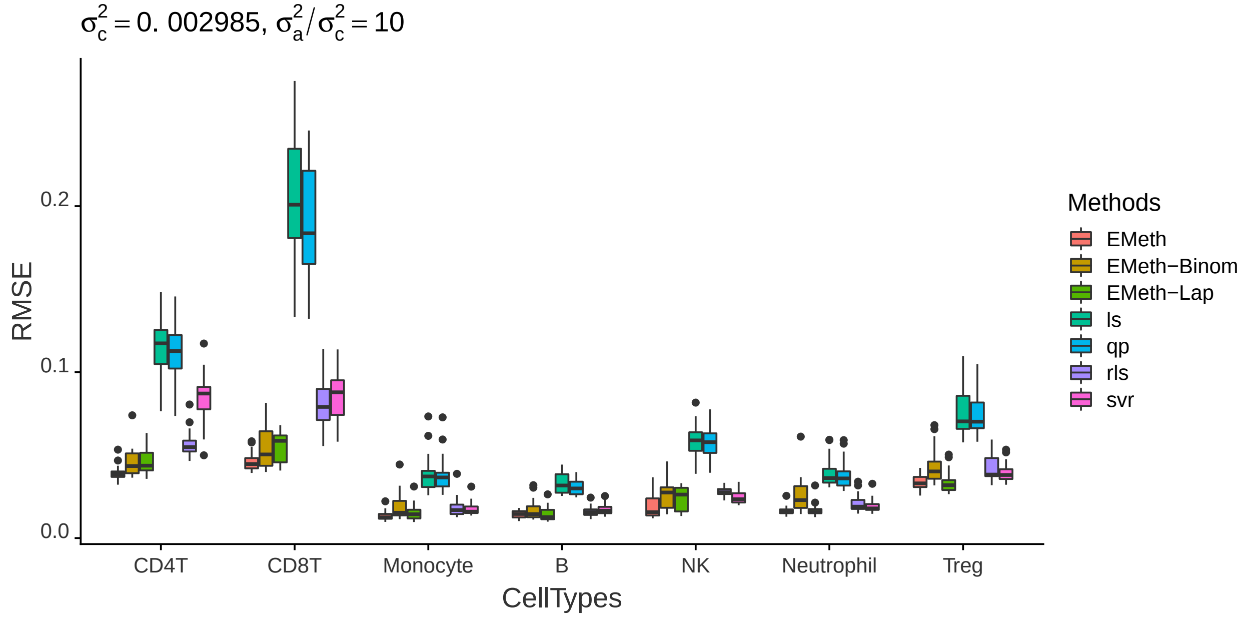

Figure S9: $\sigma^2 = 0.00298, \lambda = 10$

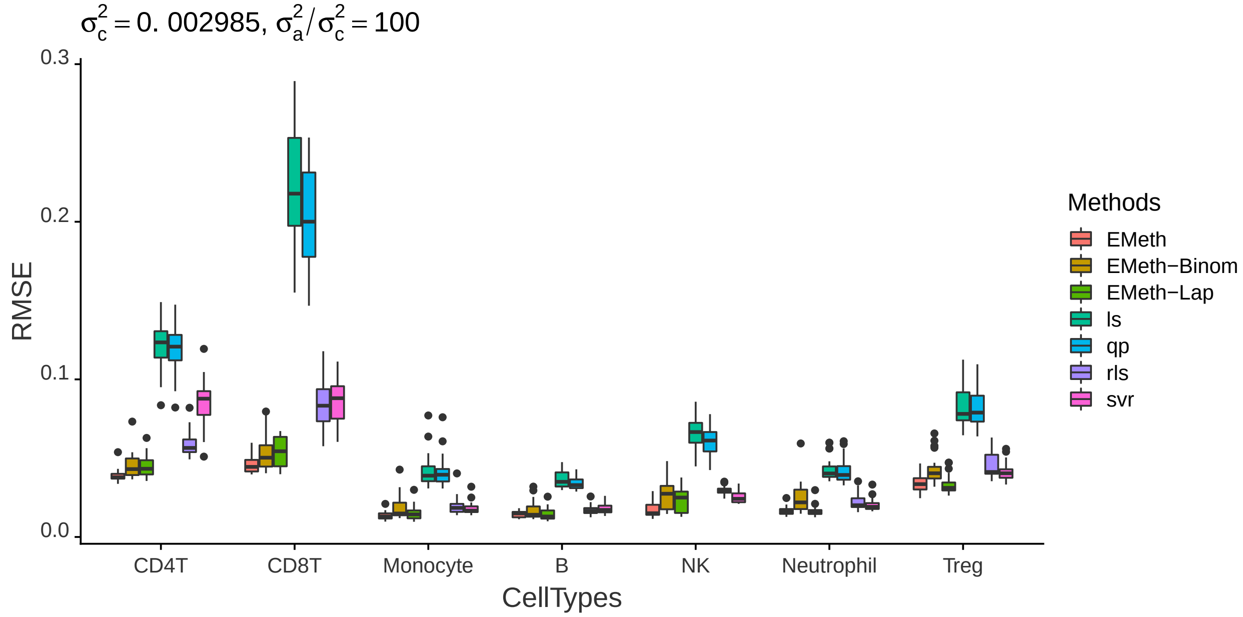

Figure S10: $\sigma^2 = 0.00298, \lambda = 100$

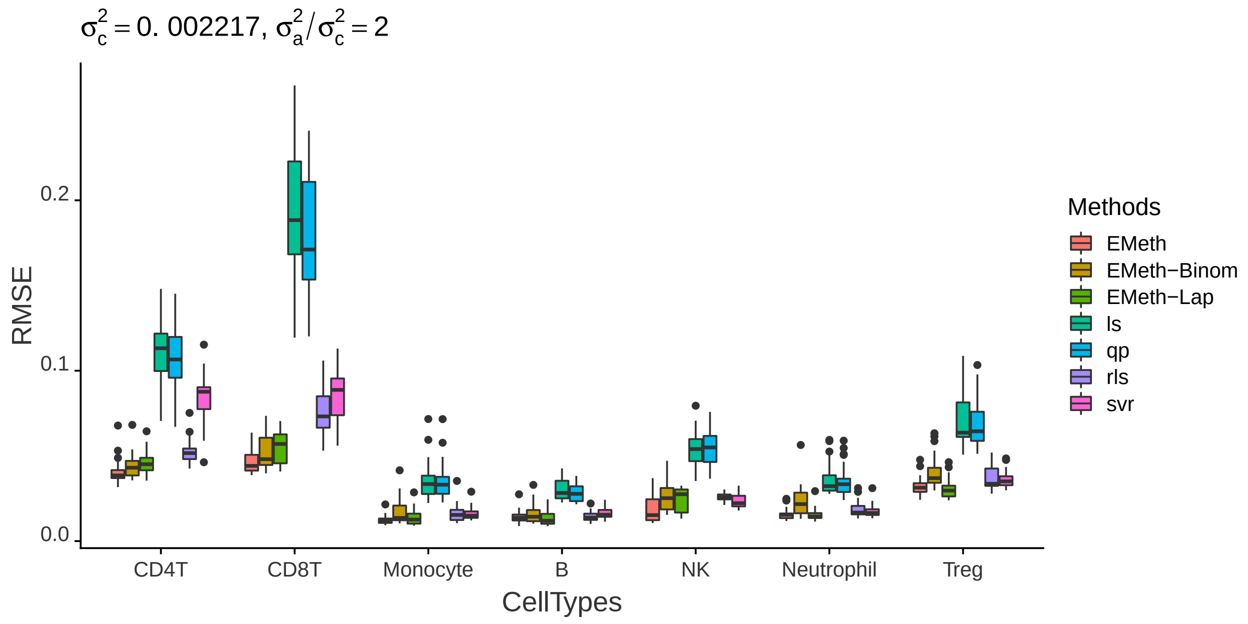

Figure S11: $\sigma^2 = 0.221, \lambda = 2$

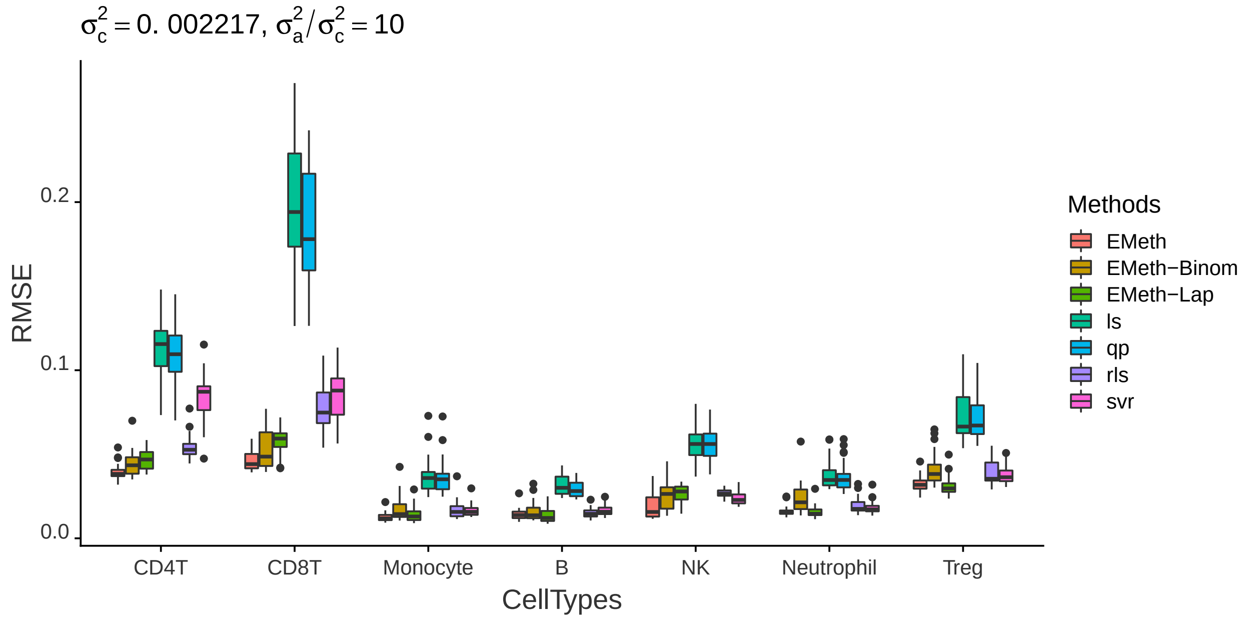

Figure S12: $\sigma^2 = 0.221, \lambda = 10$

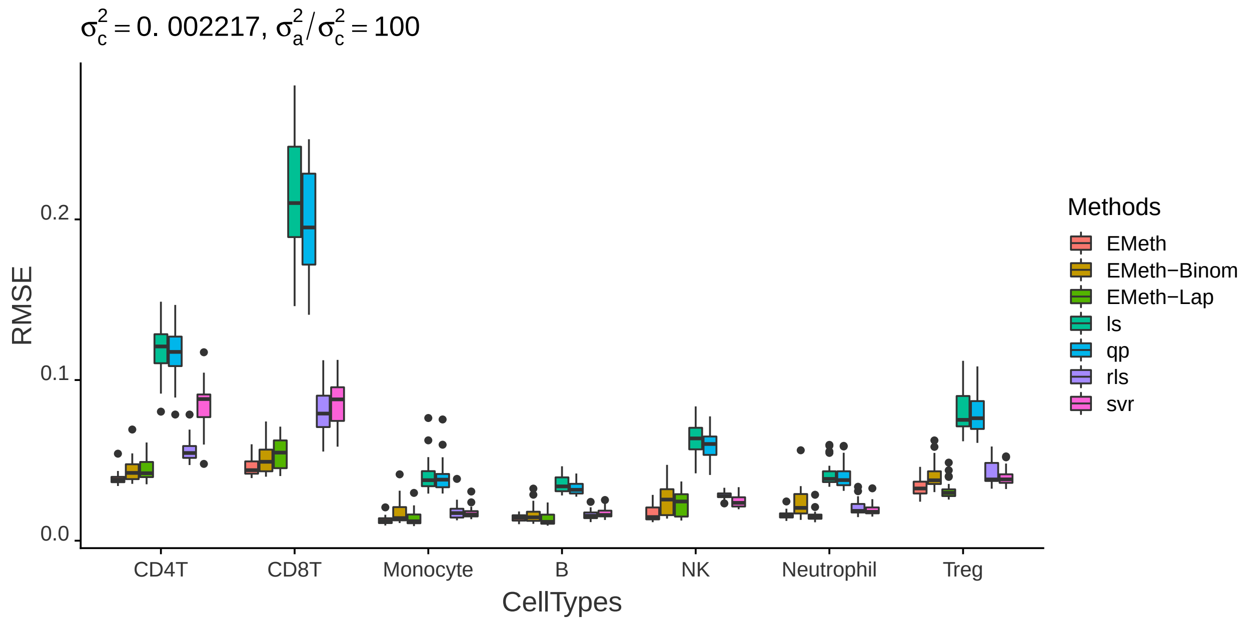

Figure S13: $\sigma^2 = 0.221, \lambda = 100$

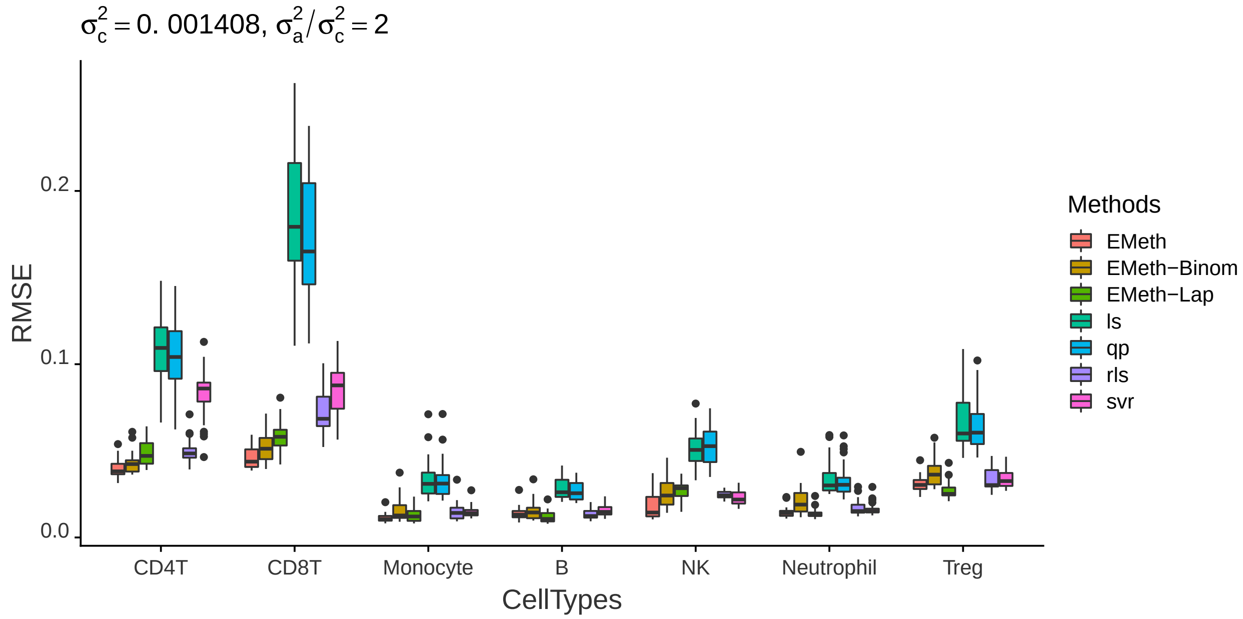

Figure S14: $\sigma^2 = 0.00141, \lambda = 2$

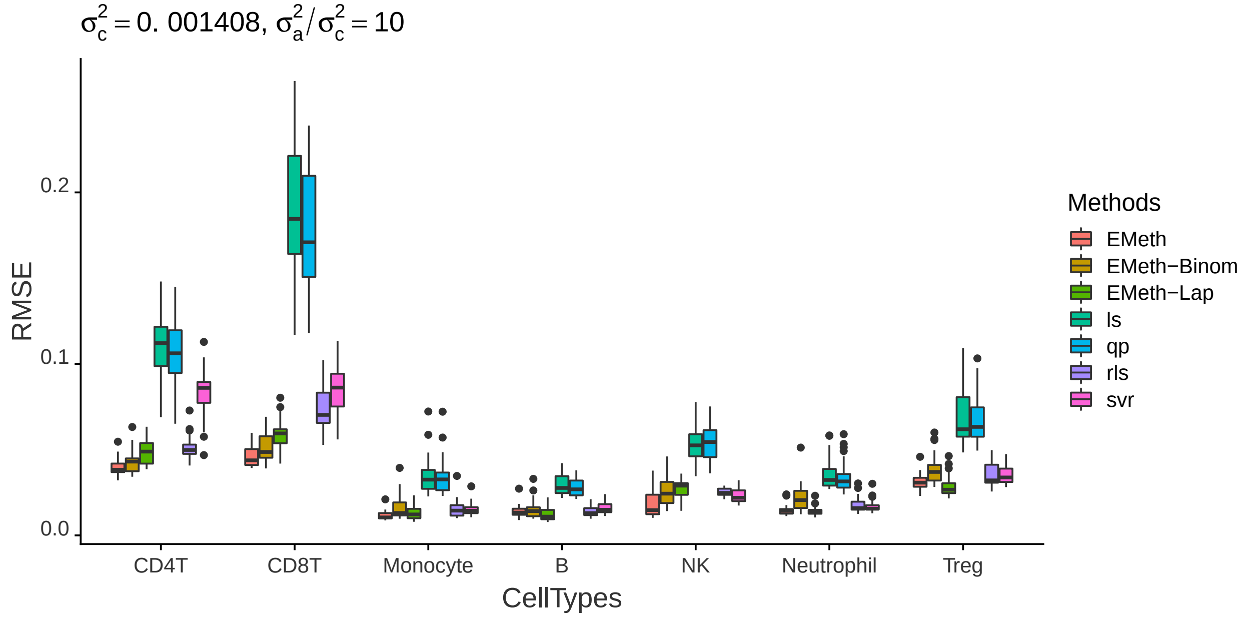

Figure S15: $\sigma^2 = 0.00141, \lambda = 10$

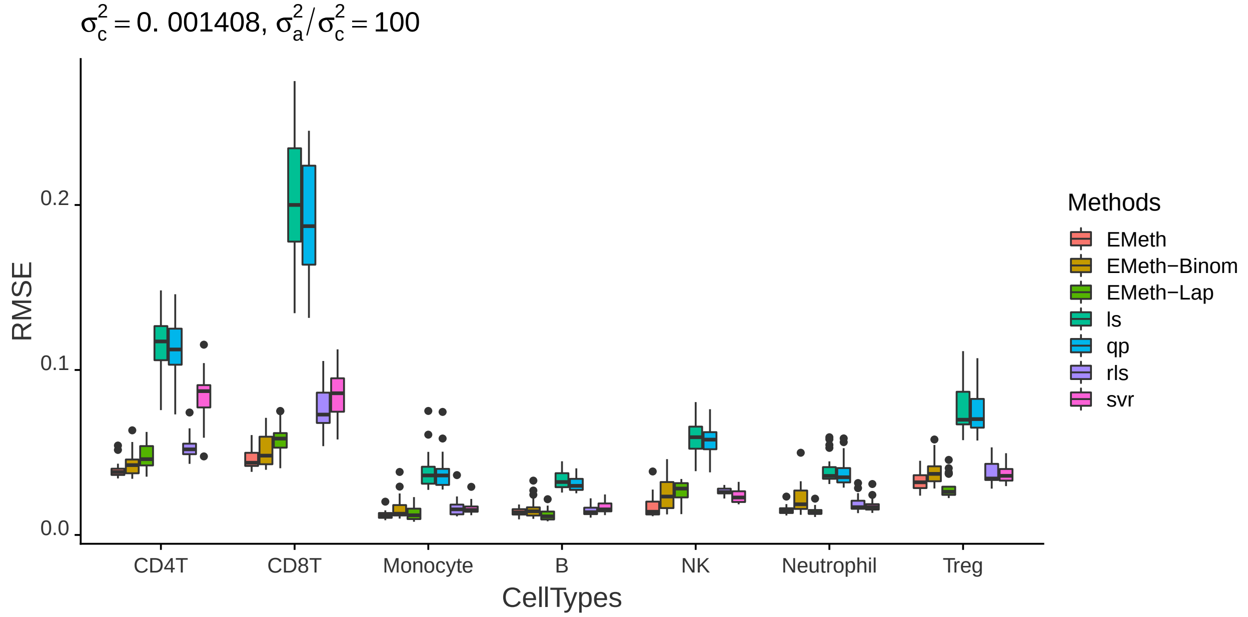

Figure S16: $\sigma^2 = 0.00141, \lambda = 100$

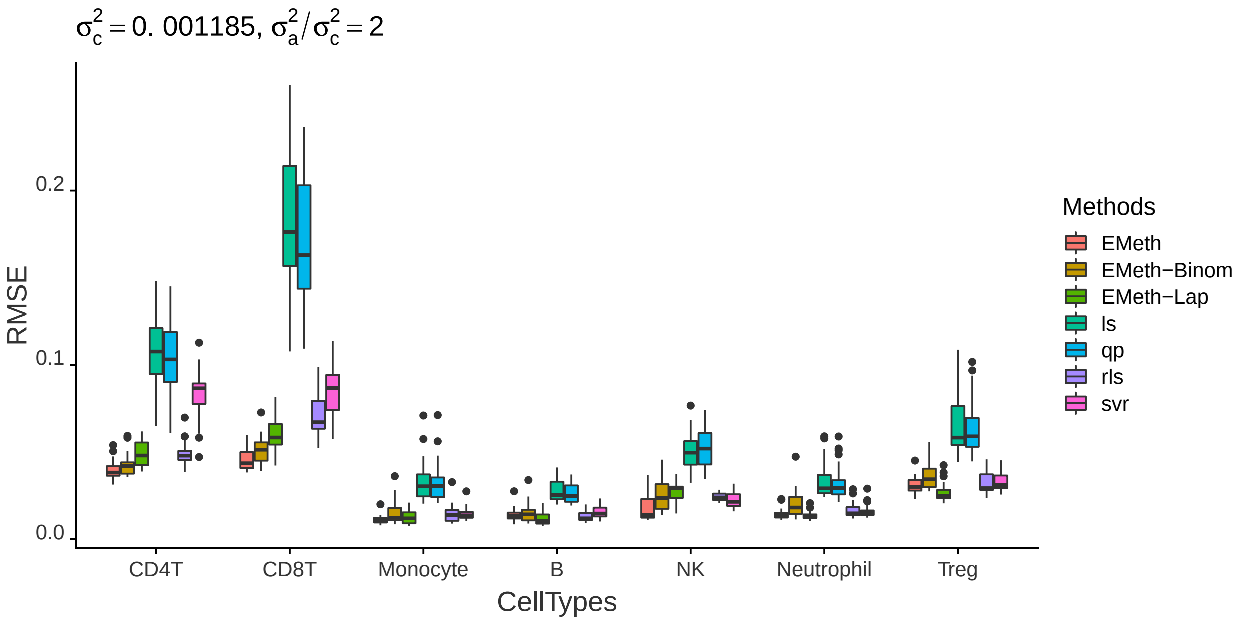

Figure S17: $\sigma^2 = 0.00118, \lambda = 2$

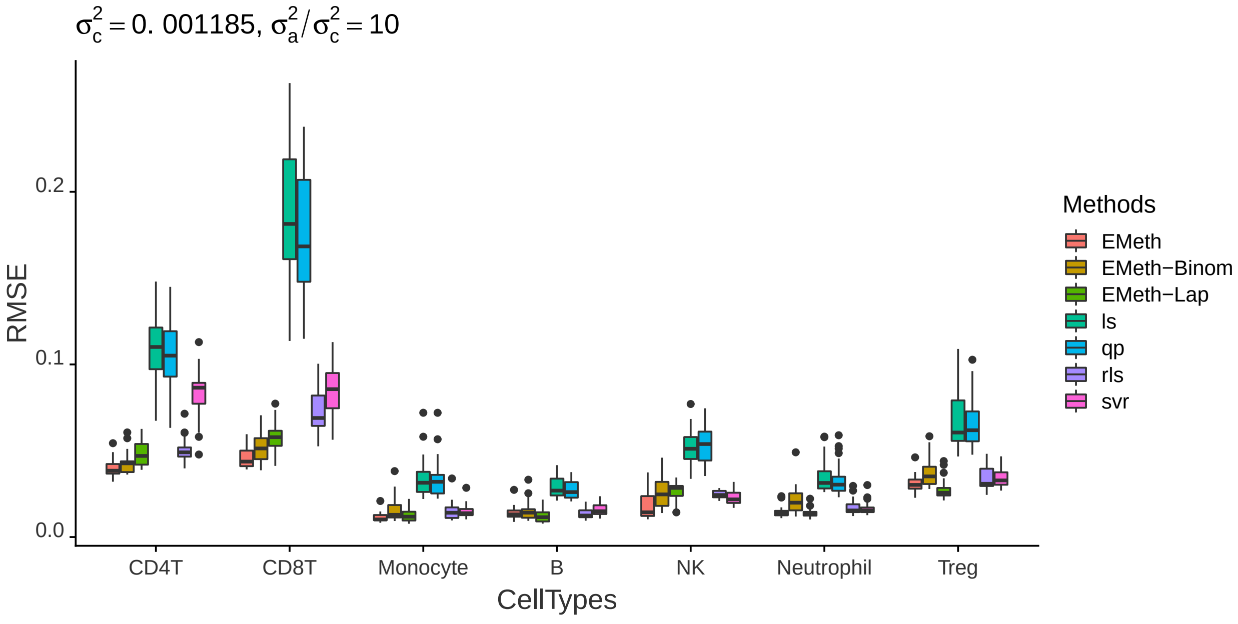

Figure S18: $\sigma^2 = 0.00118, \lambda = 10$

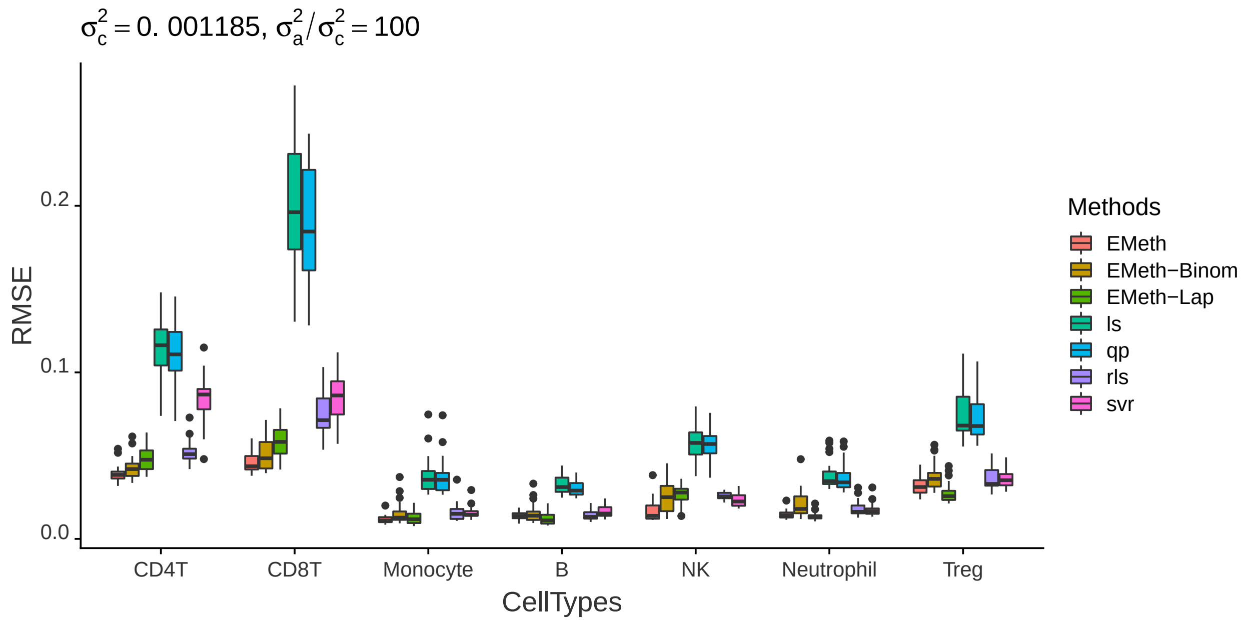

Figure S19: $\sigma^2 = 0.00118, \lambda = 100$

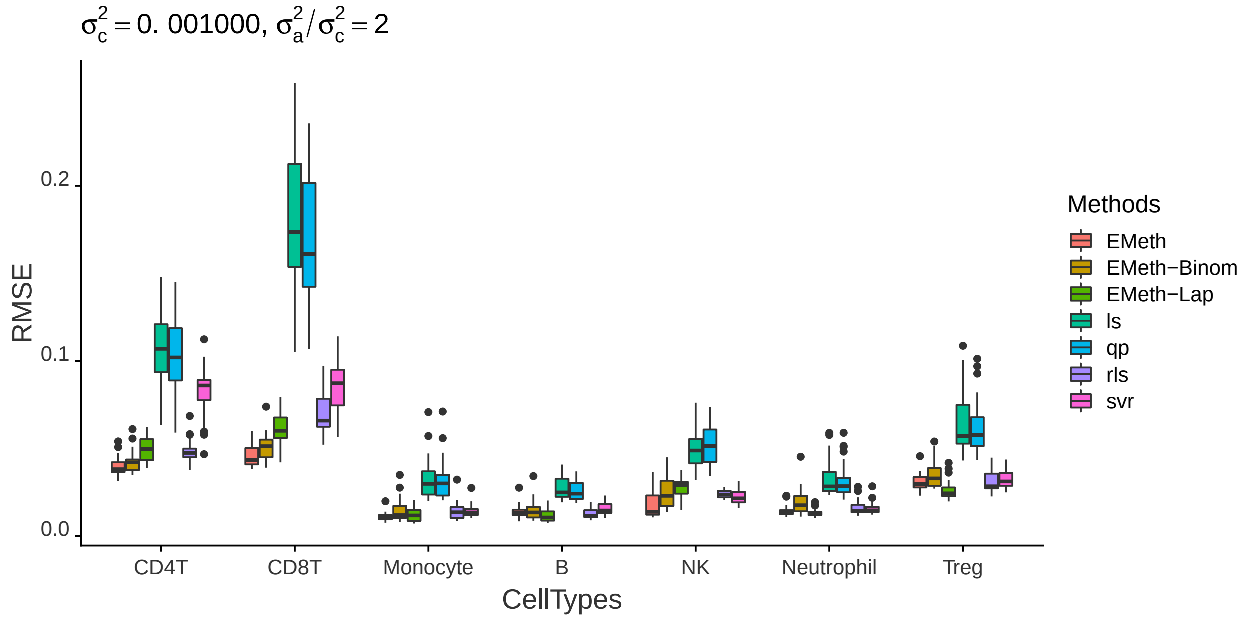

Figure S20: $\sigma^2 = 0.001, \lambda = 2$

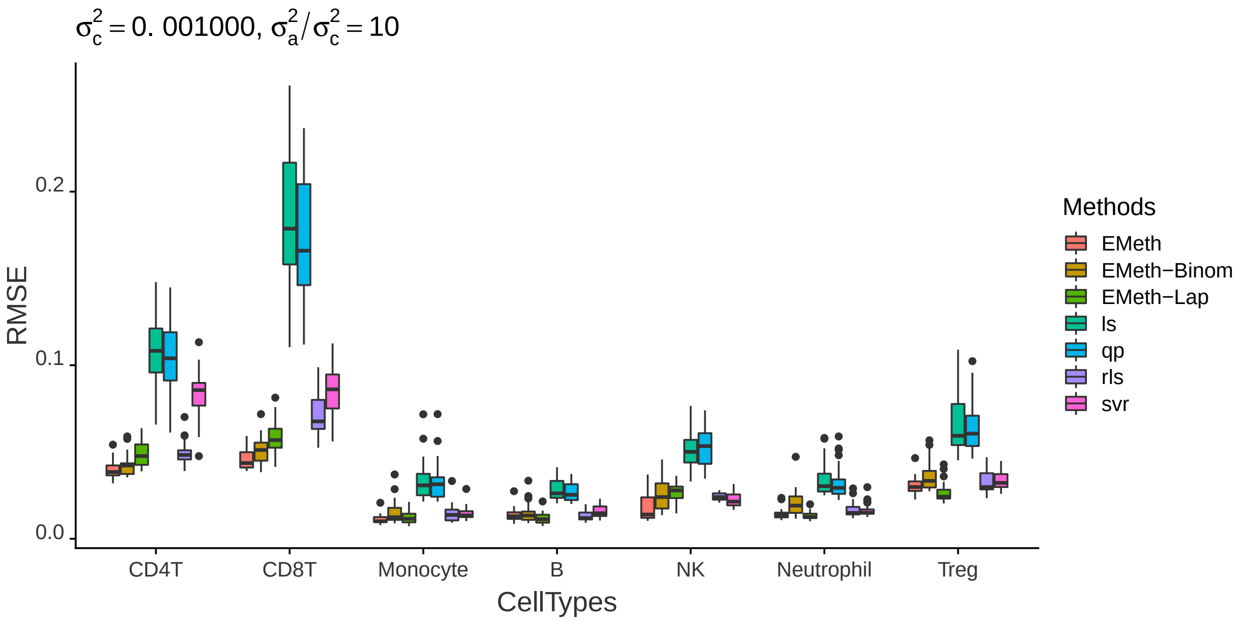

Figure S21: $\sigma^2 = 0.001, \lambda = 10$

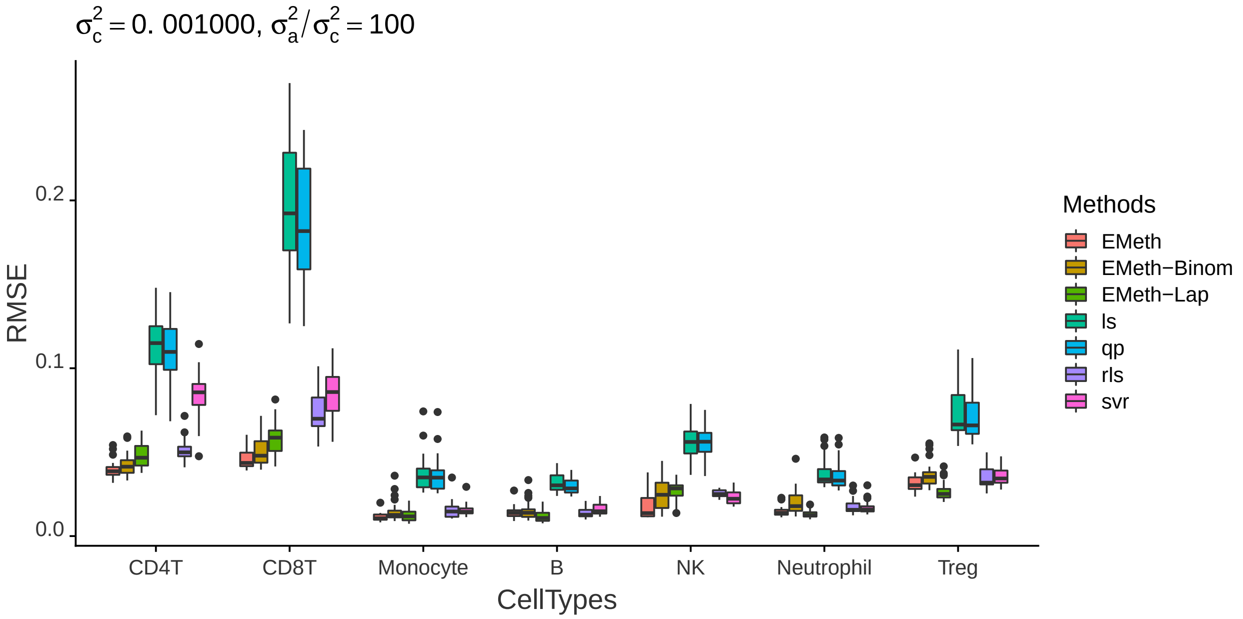

Figure S22: $\sigma^2 = 0.001, \lambda = 100$

3.3 Additional Simulation Results with larger proportion of aberrant CpG probes

The simulation results reported in the main text and previous section of this supplementary materials simulated the proportion of aberrant probes from a uniform distribution in the range of 5% to 15%. In this section, we expanded this range to 5% to 35% to evaluate the robustness of EMeth with respect to the proportion of aberrant CpG probes. As shown in the following table, EMeth still has better performance than other methods.

Table S3: Compare the performance of different methods by RMSE (average across 25 replicates) when the proportions of aberrant CpG probes are simulated from a uniform distribution in the range of 5% to 35%. Sample size is 100, and we vary two parameters, σ_c^2, which is the variance for the consistent (non-aberrant probes) and the ratio σ_a^2/σ_c^2.

σ_c^2	σ_a^2/σ_c^2	LaplaceEM	BinomEM	OriEM	svr	ls	rls	qp
	2	0.034	0.044	0.031	0.042	0.104	0.053	0.093
0.004	10	0.035	0.046	0.032	0.044	0.107	0.059	0.096
	100	0.032	0.045	0.031	0.047	0.117	0.069	0.102
	2	0.032	0.041	0.030	0.041	0.102	0.050	0.092
0.00298	10	0.033	0.044	0.031	0.043	0.105	0.055	0.094
	100	0.032	0.043	0.030	0.045	0.114	0.065	0.101
	2	0.030	0.039	0.029	0.040	0.101	0.047	0.091
0.00221	10	0.032	0.041	0.030	0.042	0.104	0.051	0.093
	100	0.030	0.041	0.029	0.044	0.112	0.060	0.099
	2	0.029	0.035	0.027	0.039	0.099	0.043	0.089
0.00141	10	0.030	0.038	0.028	0.040	0.101	0.047	0.091
	100	0.029	0.038	0.028	0.042	0.109	0.055	0.097
	2	0.029	0.035	0.026	0.038	0.098	0.042	0.089
0.00118	10	0.029	0.037	0.028	0.039	0.100	0.045	0.091
	100	0.029	0.037	0.028	0.042	0.108	0.053	0.096
	2	0.028	0.034	0.026	0.038	0.097	0.041	0.088
0.001	10	0.029	0.036	0.028	0.039	0.100	0.044	0.090
	100	0.028	0.037	0.027	0.041	0.107	0.051	0.095

4 BLUEPRINT Data

We used the cell-type specific and individual-specific DNA methylation data from the BLUEPRINT project to generate *in silico* mixtures [8]. This data set consists of three cell types: naive CD4 T cells, monocytes and neutrophils. The original data set has methylation data from 196, 197, and 133 individuals for these three cell types, respectively. We selected the individuals with DNA methylation as well as gene expression data from all three cell types and ended up with 124 individuals.

We added noise to both gene expression data and DNA methylation data. For gene expression data, we estimated the variance σ_{qk}^2 for cell type q and gene k, and then added Gaussian noise $\mathcal{N}(0, c\sigma_{qk}^2)$ to the observed gene expression data. In this study, we set c so that the performance of different methods have meaningful difference. We also added the same level of Gaussian noise relative to the observed variation to the DNA methylation data on the $M-$value scale, and then transformed them to $\beta-$values.

The noisy data were then divided to two subsets: a reference set with 56 individuals and a generation set with 68 individuals. We used the reference set to select genes for the cell type-specific gene expression reference by six types of comparisons (one versus all comparisons for all three cell types and three pairwise comparisons). For each type of comparison, we selected 100 genes with largest fold changes, among those with two sample t-test p-values $< 10^{-6}$. CpG probes were selected similarly. In total we included around 400 CpGs for DNA methylation reference and 300 genes for gene expression reference. The cell type-specific references were generated by calculating the mean DNA methylation (or mean gene expression) for each selected CpG (or gene) in each cell type across the 56 individuals in the reference set.

We generated DNA methylation (in beta value scale) of a mixture sample in the following steps. Denote the estimated standard deviation for the k-th CpG of monocyte, neutrophil, and CD4 T cell on the generation set by σ_{mk}, σ_{nk}, and σ_{tk} respectively. For the i-th individual in the generation set, denote its methylation of the k-th CpG for these three cell types by μ_{imk}, μ_{ink}, and μ_{itk}, respectively. We sampled ρ'_{im}, ρ'_{in}, and ρ'_{it} from Uniform(0,1) distribution and normalized them such that their summation equals to 1, and denoted the normalized cell type fractions as ρ_{im}, ρ_{in}, and ρ_{it}, respectively. Then we computed the mean and variance for the beta-value of the k-th CpG by

$$\mu_{ik} = \rho_{in}\mu_{ink} + \rho_{im}\mu_{imk} + \rho_{it}\mu_{itk}, \text{ and} \tag{5}$$

$$\sigma_{ik}^2 = \rho_{in}^2\sigma_{ink}^2 + \rho_{im}^2\sigma_{imk}^2 + \rho_{it}^2\sigma_{itk}^2. \tag{6}$$

Finally we generated methylation for the k-th CpG of the i-th individual by sampling from a normal distribution $\mathcal{N}(\mu_{ik}, \sigma_{ik}^2)$. To mimic the aberrant CpGs, each CpG has a probability $\pi = 0.1$ so that its variance is expanded to be $\lambda\sigma_{ik}^2$.

Similarly, we generate mixture samples for gene expression data. Note that we generate gene expression in the original scale instead of log scale of gene expression data.

5 TCGA Data

5.1 Additional results using LM22 gene expression reference

We used the preprocessed gene expression and DNA methylation data from The Pan-Cancer Atlas (PanCanAtlas) initiative `https://gdc.cancer.gov/about-data/publications/pancanatlas` [9]. We have reported the result of colon cancer (COAD) in main text. In this section we show the additional results for three other cancer types. The results in this subsection and main text is based on LM22 reference, which is the default gene expression reference of CIBERSORTx [10]. It was generated from microarray data and include 22 immune cell types.

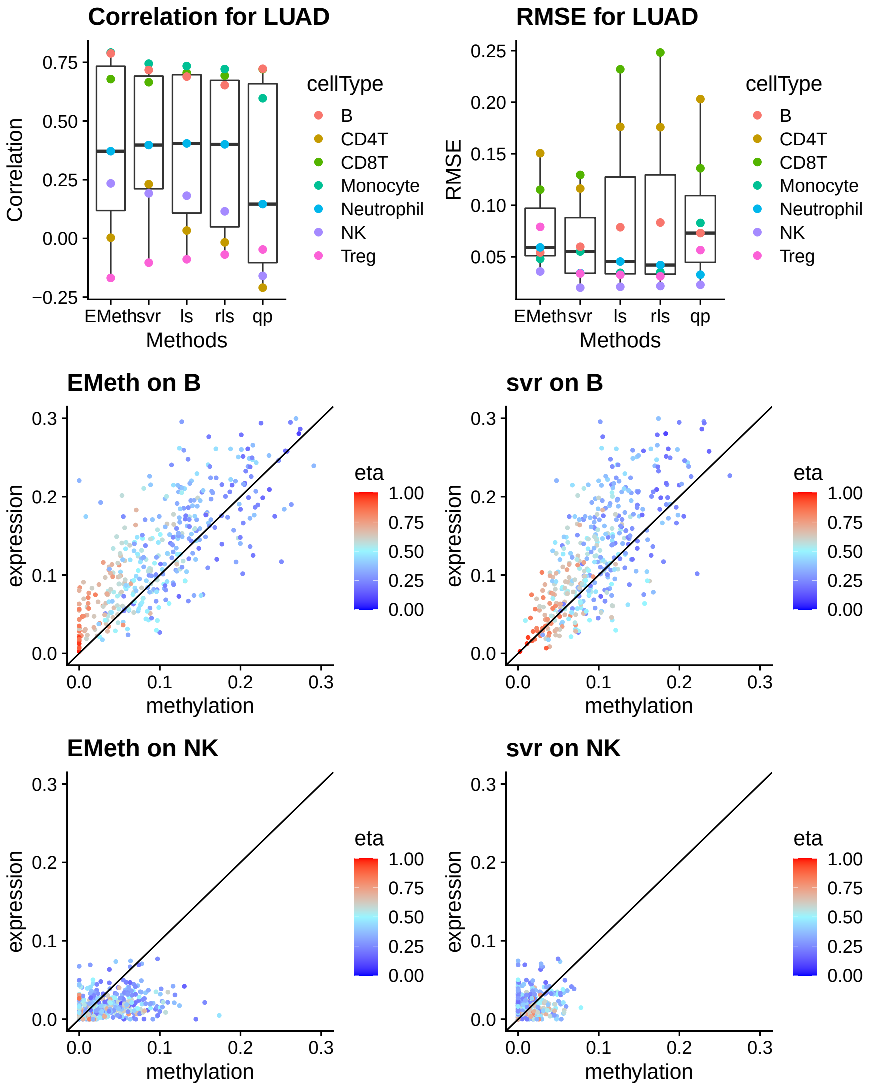

Figure S23: Additional Results for Lung Adenocarcinoma (LUAD). In the lower four panels, the points are colored by tumor purity ("eta").

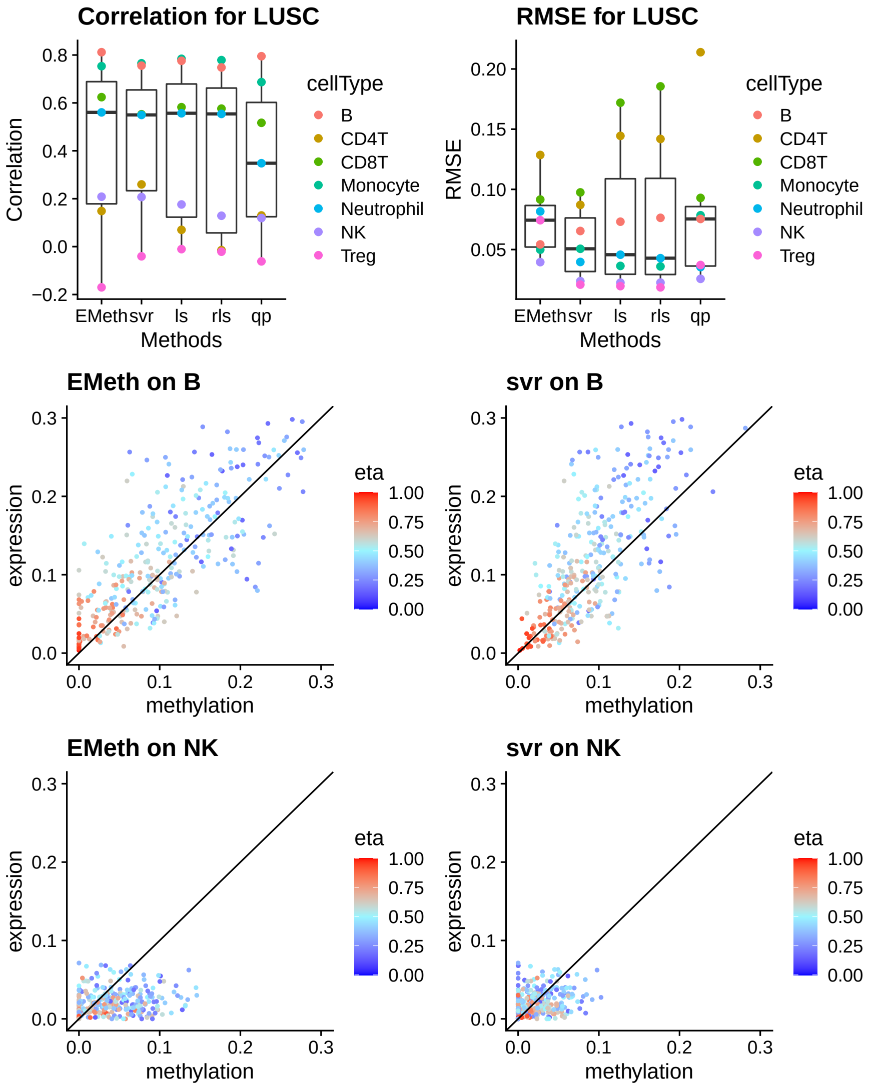

Figure S24: Additional Results for lung squamous cell carcinoma (LUSC).

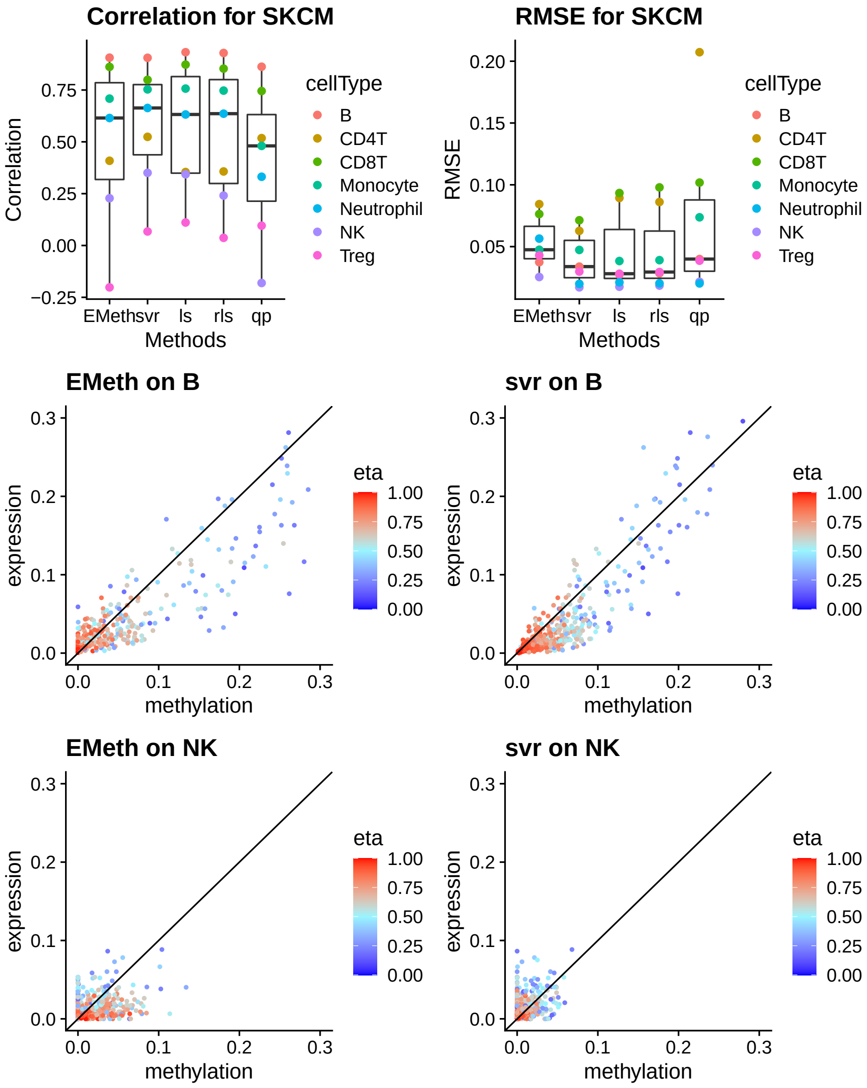

Figure S25: Additional Results for Skin Cutaneous Melanoma (SKCM).

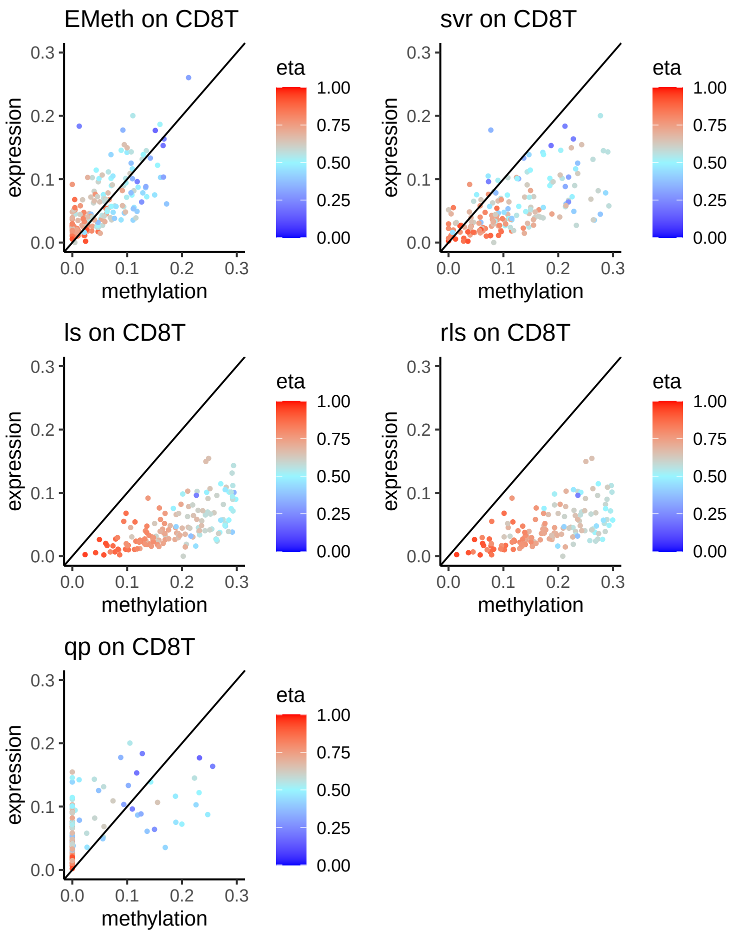

Figure S26: Compare CD8T cell type proportion estimates from gene expression (by CIBERSORTx using LM22 signatures) vs. the estimates from DNA methylation by five different methods for TCGA colon adenocarcinoma (COAD) samples. The points are colored by tumor purity ("eta").

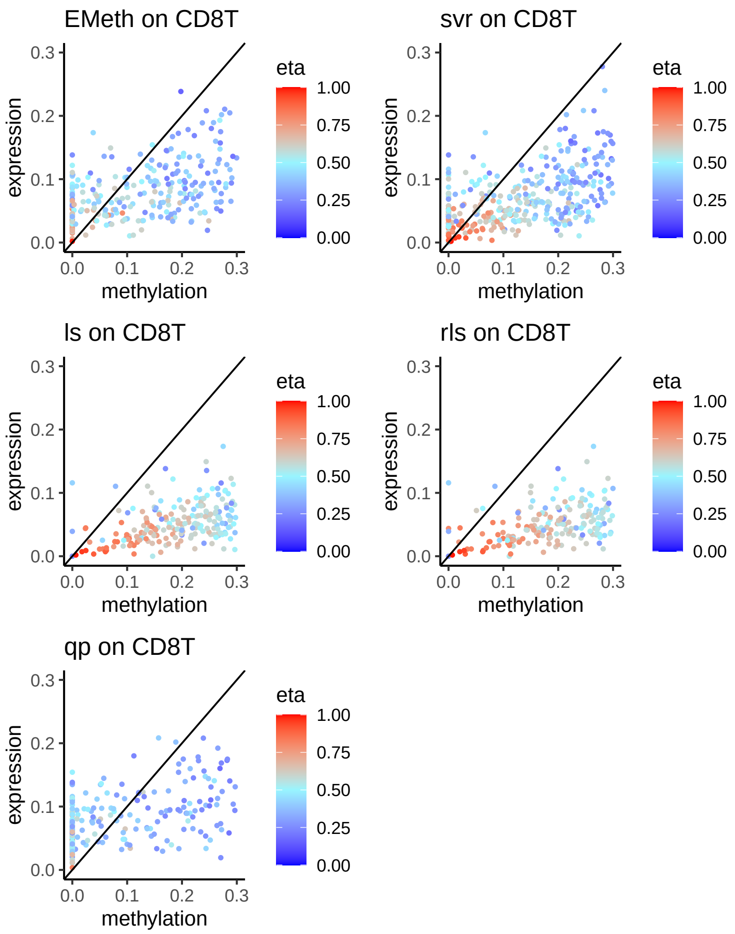

Figure S27: Compare CD8T cell type proportion estimates from gene expression (by CIBERSORTx using LM22 signatures) vs. the estimates from DNA methylation by five different methods for TCGA Lung Adenocarcinoma (LUAD) samples. The points are colored by tumor purity ("eta").

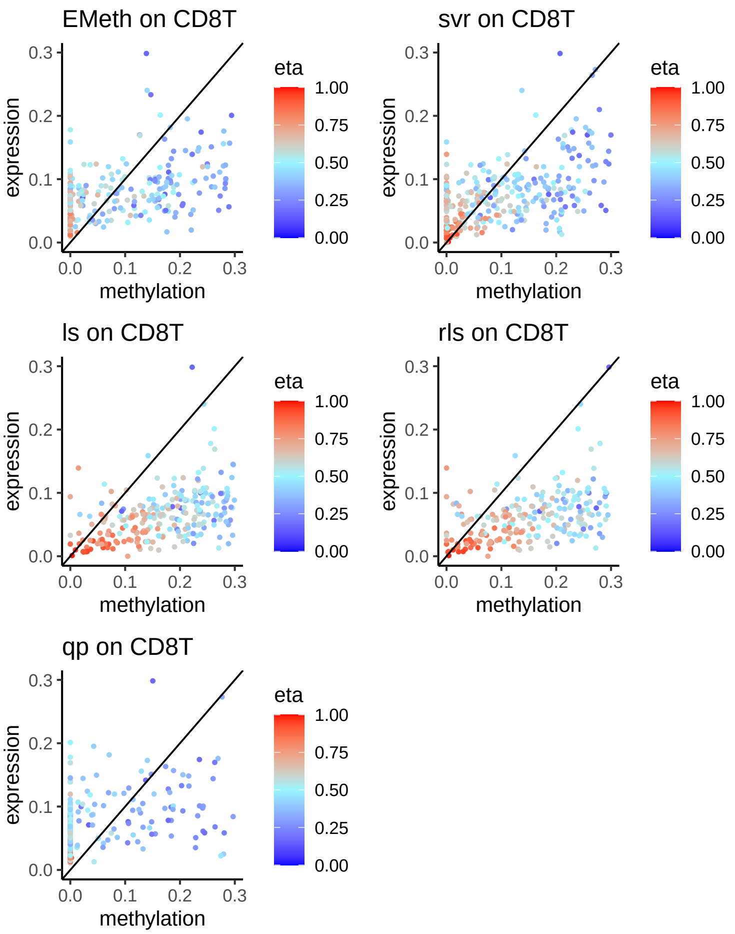

Figure S28: Compare CD8T cell type proportion estimates from gene expression (by CIBERSORTx using LM22 signatures) vs. the estimates from DNA methylation by five different methods for TCGA lung squamous cell carcinoma (LUSC) samples. The points are colored by tumor purity ("eta").

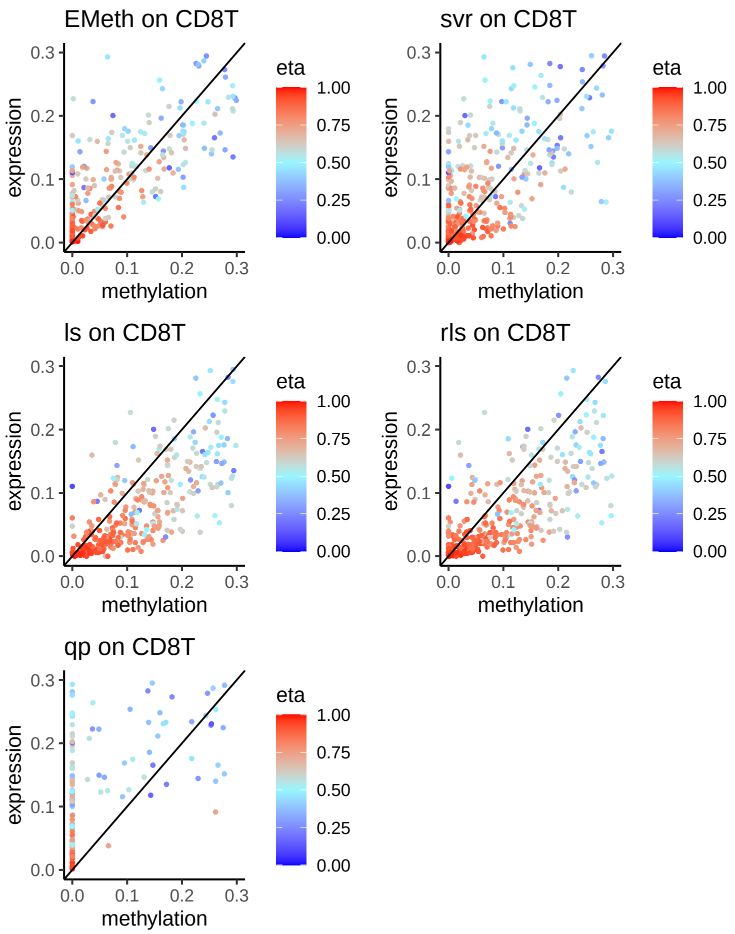

Figure S29: Compare CD8T cell type proportion estimates from gene expression (by CIBERSORTx using LM22 signatures) vs. the estimates from DNA methylation by five different methods for TCGA Skin Cutaneous Melanoma (SKCM) samples. The points are colored by tumor purity ("eta").

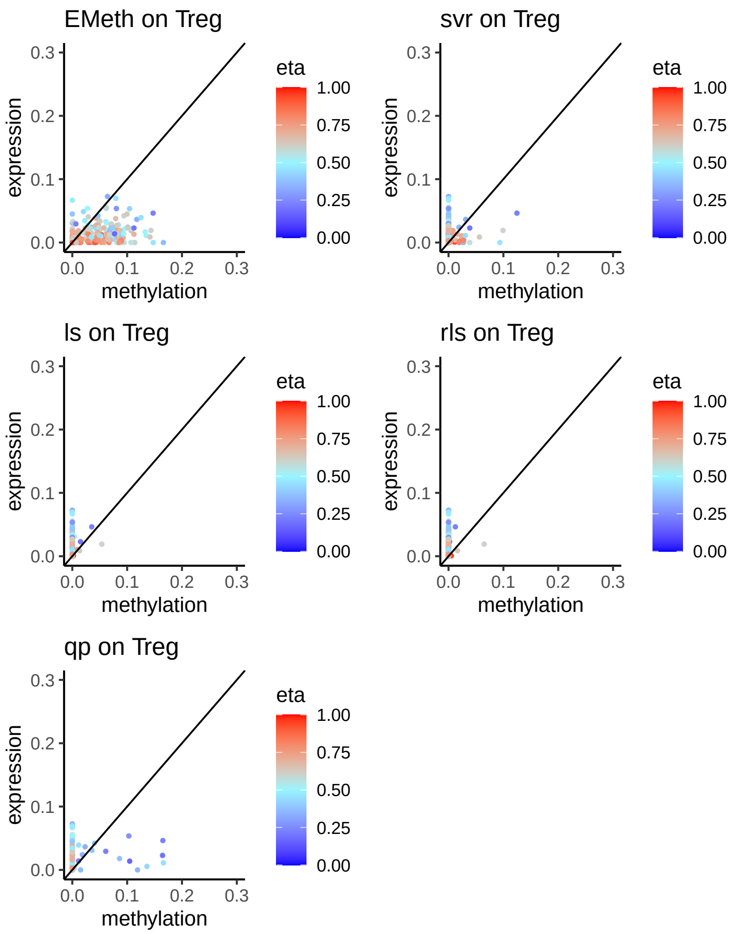

Figure S30: Compare Tregs cell type proportion estimates from gene expression (by CIBERSORTx using LM22 signatures) vs. the estimates from DNA methylation by five different methods for TCGA colon adenocarcinoma (COAD) samples.

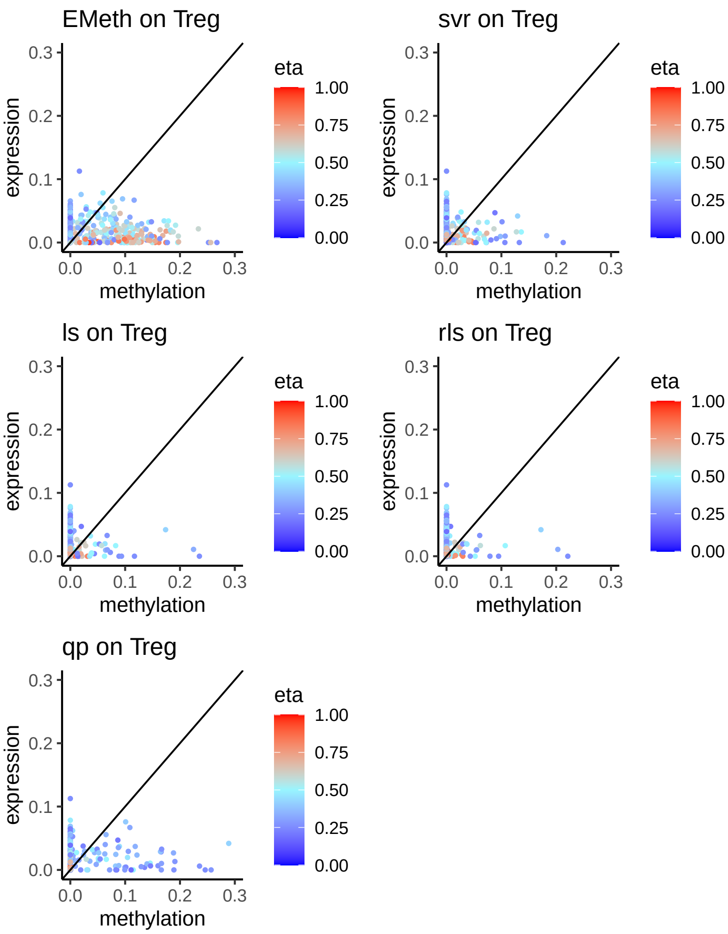

Figure S31: Compare Tregs cell type proportion estimates from gene expression (by CIBERSORTx using LM22 signatures) vs. the estimates from DNA methylation by five different methods for TCGA Lung Adenocarcinoma (LUAD) samples.

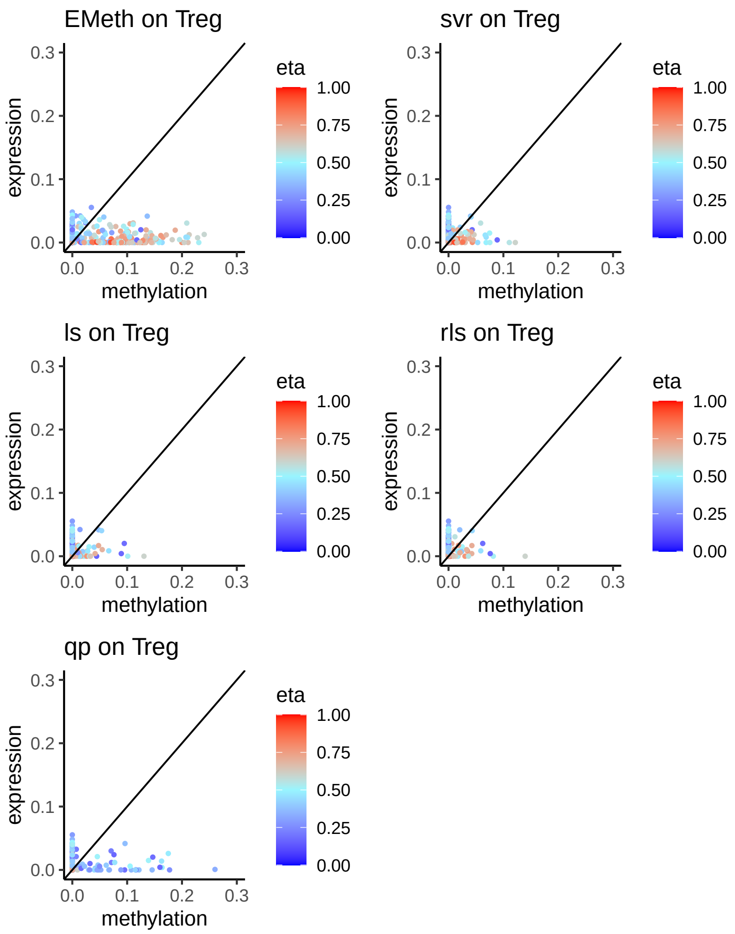

Figure S32: Compare Tregs cell type proportion estimates from gene expression (by CIBERSORTx using LM22 signatures) vs. the estimates from DNA methylation by five different methods for TCGA lung squamous cell carcinoma (LUSC) samples.

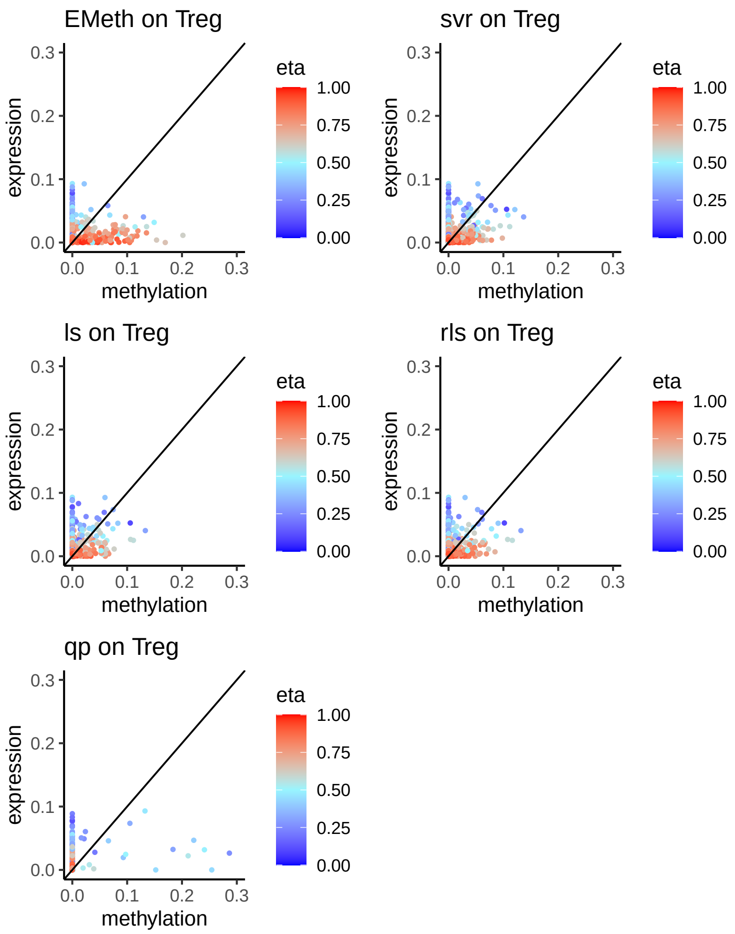

Figure S33: Compare Tregs cell type proportion estimates from gene expression (by CIBERSORTx using LM22 signatures) vs. the estimates from DNA methylation by five different methods for TCGA Skin Cutaneous Melanoma (SKCM) samples.

The following Table shows the survival analysis results in TCGA Melanoma samples, where we use Cox proportional hazard model to study the association between survival time versus age, gender, and cell type fractions. The code to generate this as well as other results are saved at `https://github.com/Sun-lab/dMeth/blob/master/4_cellType_asso.Rmd`.

Table S4: Association between cell type fractions and survival time in TCGA Melanoma samples

	gene-expression-based estimates				DNA-methylation-based estimates			
Cell Type	beta	HR	95% CI for HR	p.value	beta	HR	95% CI for HR	p.value
CD4T	-0.147	0.86	(0.77–0.96)	0.0086	-0.012	0.99	(0.91–1.07)	0.77
CD8T	-0.160	0.85	(0.78–0.93)	2.0e-04	-0.135	0.87	(0.82–0.93)	6.3e-05
Monocyte	-0.099	0.91	(0.80–1.02)	0.11	-0.039	0.96	(0.89–1.04)	0.35
B	-0.102	0.90	(0.82–1.00)	0.053	-0.127	0.88	(0.81–0.96)	0.003
NK	-0.054	0.95	(0.81–1.11)	0.5	-0.066	0.94	(0.84–1.05)	0.25
Neutrophil	-0.003	1.00	(0.83–1.19)	0.97	0.111	1.12	(1.02–1.22)	0.016
Treg	-0.197	0.82	(0.72–0.93)	0.0026	0.115	1.12	(1.02–1.23)	0.013

5.2 Additional results using SF11 gene expression reference

We generated another cell type-specific gene expression reference for melanoma tumor samples using single cell RNA-seq (scRNA-seq) data from Sade-Feldman (2018) [11]. They collected 16,291 individual immune cells from 48 tumor samples of melanoma patients and their clustering analysis identified 11 clusters. We chose this dataset for two reasons. One is that these cells were collected from melanoma tumor samples, and thus a good match to the bulk melanoma tumor samples. The other reason is that this scRNA-seq dataset were generated using SMART-seq2 protocol, which provide full coverage of each transcript, matching the bulk RNA-seq data. In contrast, another popular scRNA-seq protocol Drop-seq (e.g., 10x Genomics) only measures the gene expression on the 3' or 5' of each transcript. We downloaded the scRNA-seq data from `https://www.ncbi.nlm.nih.gov/geo/query/acc.cgi?acc=GSE120575`, and clustered the cells using a popular pipeline that consists of 3 steps: identifying genes with high variance conditioning on their mean values, calculating PCs using those genes, and then performing K-means clustering using top PCs. Our clustering results are highly consistent with the clustering results reported by Sade-Feldman (2018) [11]. We classified those cells whose cluster membership were consistent between our results and the results of Sade-Feldman (2018) [11] into 11 cell types, and conducted differential expression analysis [12] to identify a total of 594 marker genes with cell type-specific expression. We refer to this signature matrix of 594 genes for 11 cell types as SF11. More details of clustering and signature gene selection can be found at `https://github.com/Sun-lab/IT-predictor/tree/master/R/SF2018_analysis`.

Using this SF11 signature, we applied CIBERSORTx to estimate cell type proportions of TCGA SKCM samples, and collapsed a few of these 11 cell types to match the cell types from methylation data. Then we compared the three cell proportion estimates: two were estimated using gene expression by CIBERSORTx with LM22 or SF11 signature, respectively, and one was estimated using DNA methylation data by EMeth. We summarize the results using the following figures. The complete pipeline and results can be found at `https://github.com/Sun-lab/dMeth/blob/master/TCGA_pipeline/4_SKCM_ct_prop_comparison.Rmd`.

First, as a sanity check, we confirmed that the gene expression from the Sade-Feldman (2018) data have reasonable correlation with the gene expression from the LM22 signature matrix of CIBERSORT. LM22 signature matrix has 547 genes, among which 323 genes have sufficient gene expression in this scRNA-seq dataset. The expression of these 323 have moderate to strong correlations between the LM22 matrix and this scRNA-seq data set (Figure S34). Note that we did not use these genes to construct our SF11 signature matrix, instead, we ran differential expression analysis and identified genes that were differentially expressed in the scRNA-seq data. The expression of a few marker genes included in the SF11 matrix are illustrated in Figures S35-S40. Finally, we estimated cell type proportions in the TCGA SKCM samples and compared the three versions of cell proportion estimates: those estimates based on gene expression LM22 signature or SF11 signature or those estimates based on DNA methylation (by EMeth). (Figures S41).

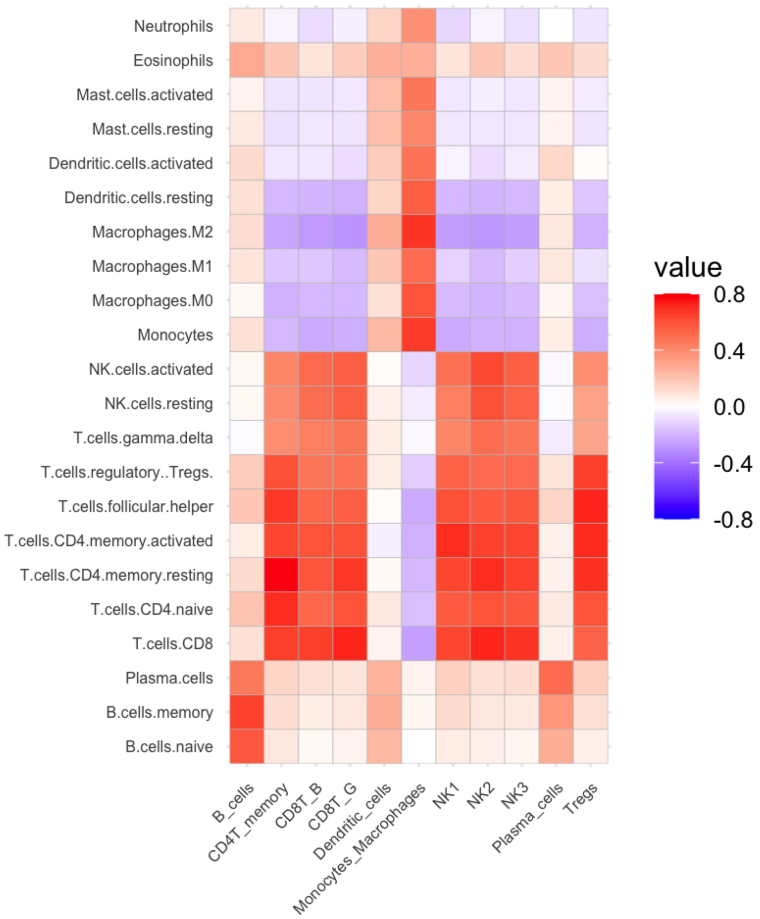

Figure S34: The correlation of gene expression from the scRNA-seq data from Sade-Feldman (2018) [11] (x-axis) and the LM22 signature (y-axis).

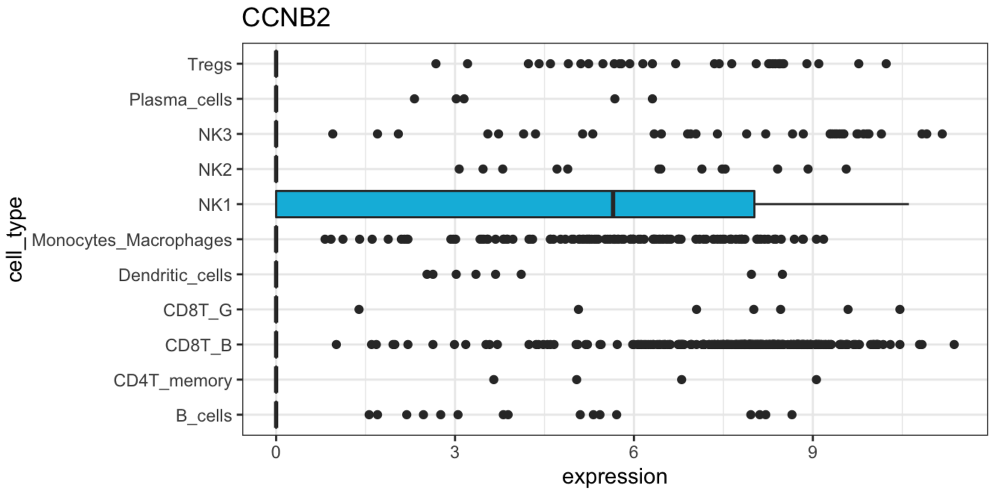

Figure S35: Gene expression of CCNB2 in different cell types.

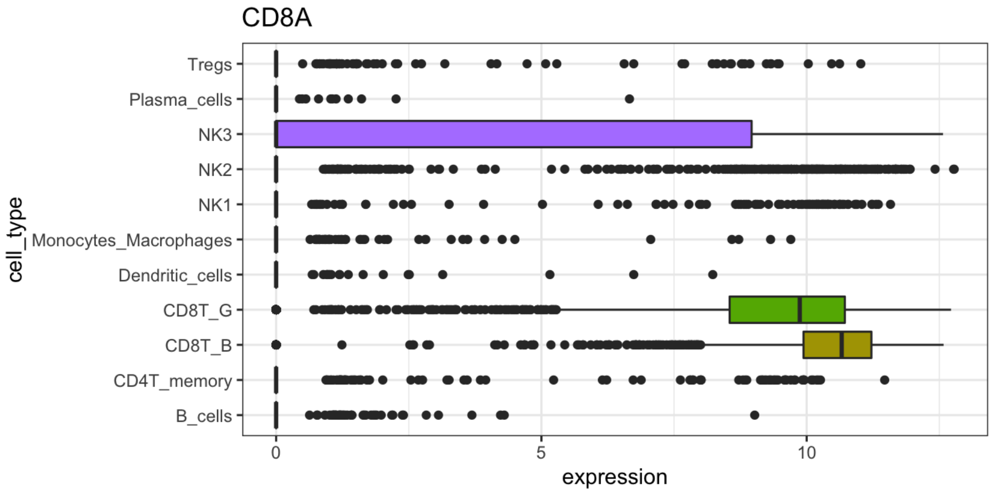

Figure S36: Gene expression of CD8A in different cell types.

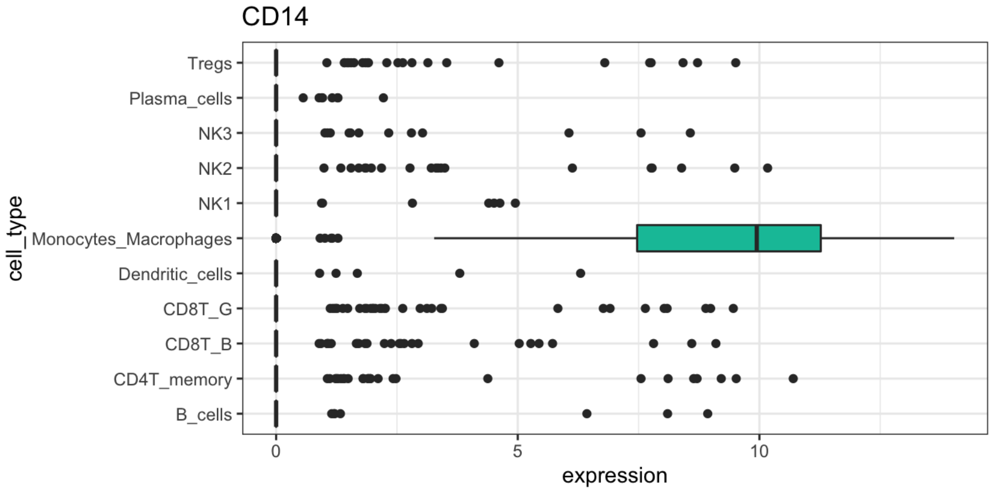

Figure S37: Gene expression of CD14 in different cell types.

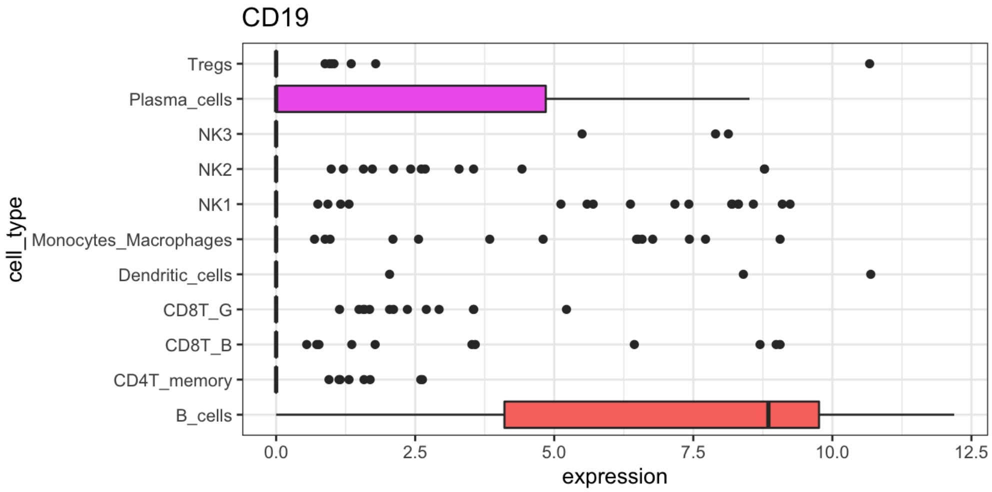

Figure S38: Gene expression of CD19 in different cell types.

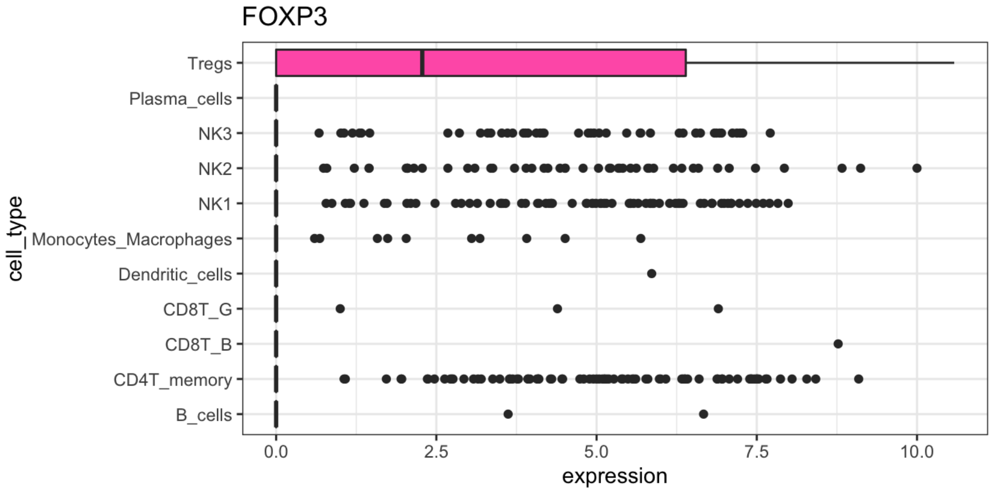

Figure S39: Gene expression of FOXP3 in different cell types.

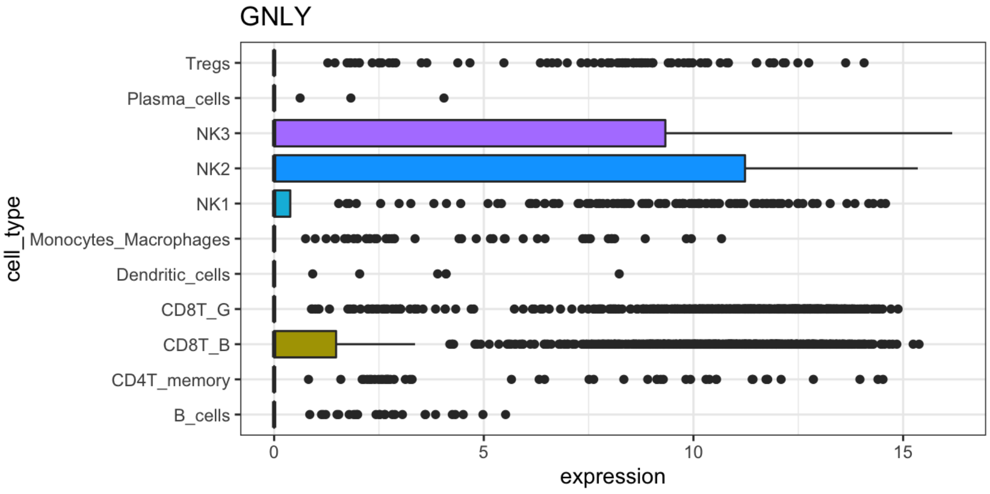

Figure S40: Gene expression of GNLY in different cell types.

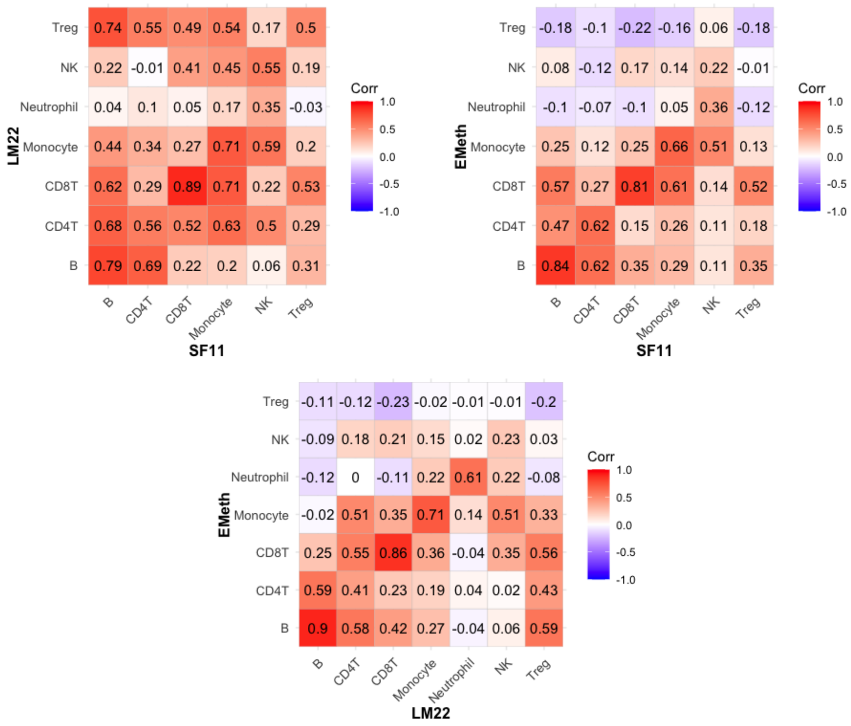

Figure S41: Correlation matrix (Pearson correlation) of cell type proportion estimates based gene expression LM22 signature matrix, gene expression SF11 signature matrix, or DNA methylation of 7 cell types used by EMeth.

References

[1] Lovisa E. Reinius, Nathalie Acevedo, Maaike Joerink, Göran Pershagen, Sven-Erik Dahlén, Dario Greco, Cilla Söderhäll, Annika Scheynius, and Juha Kere. Differential DNA methylation in purified human blood cells: implications for cell lineage and studies on disease susceptibility. *PloS one*, 7(7):e41361–e41361, 2012. 22848472[pmid].

[2] Yuxia Zhang, Jovana Maksimovic, Gaetano Naselli, Junyan Qian, Michael Chopin, Marnie E. Blewitt, Alicia Oshlack, and Leonard C. Harrison. Genome-wide DNA methylation analysis identifies hypomethylated genes regulated by foxp3 in human regulatory t cells. *Blood*, 122(16):2823–2836, Oct 2013. 23974203[pmid].

[3] Lindsay M. Reynolds, Jackson R. Taylor, Jingzhong Ding, Kurt Lohman, Craig Johnson, David Siscovick, Gregory Burke, Wendy Post, Steven Shea, David R. Jr Jacobs, Hendrik Stunnenberg, Stephen B. Kritchevsky, Ina Hoeschele, Charles E. McCall, David Herrington, Russell P. Tracy, and Yongmei Liu. Age-related variations in the methylome associated with gene expression in human monocytes and t cells. *Nature communications*, 5:5366–5366, Nov 2014. 25404168[pmid].

[4] Marta Kulis, Angelika Merkel, Simon Heath, Ana C. Queirós, Ronald P. Schuyler, Giancarlo Castellano, Renée Beekman, Emanuele Raineri, Anna Esteve, Guillem Clot, Néria Verdaguer-Dot, Martí Duran-Ferrer, Nuria Russiñol, Roser Vilarrasa-Blasi, Simone Ecker, Vera Pancaldi, Daniel Rico, Lidia Agueda, Julie Blanc, David Richardson, Laura Clarke, Avik Datta, Marien Pascual, Xabier Agirre, Felipe Prosper, Diego Alignani, Bruno Paiva, Gersende Caron, Thierry Fest, Marcus O. Muench, Marina E. Fomin, Seung-Tae Lee, Joseph L. Wiemels, Alfonso Valencia, Marta Gut, Paul Flicek, Hendrik G. Stunnenberg, Reiner Siebert, Ralf Küppers, Ivo G. Gut, Elías Campo, and José I. Martín-Subero. Whole-genome fingerprint of the DNA methylome during human b cell differentiation. *Nature genetics*, 47(7):746–756, Jul 2015. 26053498[pmid].

[5] Heinrich Schlums, Frank Cichocki, Bianca Tesi, Jakob Theorell, Vivien Beziat, Tim D. Holmes, Hongya Han, Samuel C. C. Chiang, Bree Foley, Kristin Mattsson, Stella Larsson, Marie Schaffer, Karl-Johan Malmberg, Hans-Gustaf Ljunggren, Jeffrey S. Miller, and Yenan T. Bryceson. Cytomegalovirus infection drives adaptive epigenetic diversification of nk cells with altered signaling and effector function. *Immunity*, 42(3):443–456, Mar 2015. 25786176[pmid].

[6] Patrick Coit, Srilakshmi Yalavarthi, Mikhail Ognenovski, Wenpu Zhao, Sarfaraz Hasni, Jonathan D. Wren, Mariana J. Kaplan, and Amr H. Sawalha. Epigenome profiling reveals significant DNA demethylation of interferon signature genes in lupus neutrophils. *Journal of autoimmunity*, 58:59–66, Apr 2015. 25638528[pmid].

[7] Benjamin M Bolstad, Rafael A Irizarry, Magnus Åstrand, and Terence P. Speed. A comparison of normalization methods for high density oligonucleotide array data based on variance and bias. *Bioinformatics*, 19(2):185–193, 2003.

[8] Lu Chen, Bing Ge, Francesco Paolo Casale, Louella Vasquez, Tony Kwan, Diego Garrido-Martín, Stephen Watt, Ying Yan, Kousik Kundu, Simone Ecker, Avik Datta, David Richardson, Frances Burden, Daniel Mead, Alice L. Mann, Jose Maria Fernandez, Sophia Rowlston, Steven P. Wilder, Samantha Farrow, Xiaojian Shao, John J. Lambourne, Adriana Redensek, Cornelis A. Albers, Vyacheslav Amstislavskiy, Sofie Ashford, Kim Berentsen, Lorenzo Bomba, Guillaume Bourque, David Bujold, Stephan Busche, Maxime Caron, Shu-Huang Chen, Warren Cheung, Oliver Delaneau, Emmanouil T. Dermitzakis, Heather Elding, Irina Colgiu, Frederik O. Bagger, Paul Flicek, Ehsan Habibi, Valentina Iotchkova, Eva Janssen-Megens, Bowon Kim, Hans Lehrach, Ernesto Lowy, Amit Mandoli, Filomena Matarese, Matthew T. Maurano, John A. Morris, Vera Pancaldi, Farzin Pourfarzad, Karola Rehnstrom, Augusto Rendon, Thomas Risch, Nilofar Sharifi, Marie-Michelle Simon, Marc Sultan, Alfonso Valencia, Klaudia Walter, Shuang-Yin Wang, Mattia Frontini, Stylianos E. Antonarakis, Laura Clarke, Marie-Laure Yaspo, Stephan Beck, Roderic Guigo, Daniel Rico, Joost H. A. Martens, Willem H. Ouwehand, Taco W. Kuijpers, Dirk S. Paul, Hendrik G. Stunnenberg, Oliver Stegle, Kate Downes, Tomi Pastinen, and Nicole Soranzo. Genetic

drivers of epigenetic and transcriptional variation in human immune cells. *Cell*, 167(5):1398–1414.e24, Nov 2016. 27863251[pmid].

[9] Katherine A Hoadley, Christina Yau, Toshinori Hinoue, Denise M Wolf, Alexander J Lazar, Esther Drill, Ronglai Shen, Alison M Taylor, Andrew D Cherniack, Vésteinn Thorsson, et al. Cell-of-origin patterns dominate the molecular classification of 10,000 tumors from 33 types of cancer. *Cell*, 173(2):291–304, 2018.

[10] Aaron M Newman, Chloé B Steen, Chih Long Liu, Andrew J Gentles, Aadel A Chaudhuri, Florian Scherer, Michael S Khodadoust, Mohammad S Esfahani, Bogdan A Luca, David Steiner, et al. Determining cell type abundance and expression from bulk tissues with digital cytometry. *Nature biotechnology*, 37(7):773–782, 2019.

[11] Moshe Sade-Feldman, Keren Yizhak, Stacey L Bjorgaard, John P Ray, Carl G de Boer, Russell W Jenkins, David J Lieb, Jonathan H Chen, Dennie T Frederick, Michal Barzily-Rokni, et al. Defining t cell states associated with response to checkpoint immunotherapy in melanoma. *Cell*, 175(4):998–1013, 2018.

[12] Greg Finak, Andrew McDavid, Masanao Yajima, Jingyuan Deng, Vivian Gersuk, Alex K Shalek, Chloe K Slichter, Hannah W Miller, M Juliana McElrath, Martin Prlic, et al. Mast: a flexible statistical framework for assessing transcriptional changes and characterizing heterogeneity in single-cell rna sequencing data. *Genome biology*, 16(1):1–13, 2015.
